# A meta-analysis of bone conduction 80 Hz auditory steady-state response thresholds in adults and infants

**DOI:** 10.3389/fauot.2026.1837322

**Published:** 2026-06-19

**Authors:** Emanuele Perugia, Constantina Georga

**Affiliations:** 1 Manchester Centre for Audiology and Deafness, Division of Psychology, Communication & Human Neuroscience, School of Health Sciences, Faculty of Biology, Medicine and Health, The University of Manchester, Manchester, United Kingdom; 2 Royal Berkshire NHS Foundation Trust, Reading, United Kingdom

**Keywords:** auditory steady-state response, bone conduction, hearing threshold, meta-analysis, objective audiometry

## Abstract

**Introduction:**

Auditory steady-state responses (ASSRs) objectively estimate hearing thresholds in individuals unable to provide behavioral responses. Bone conduction (BC) testing differentiates conductive from sensorineural hearing loss. Accurate BC ASSR threshold estimation relies on correction values, which are not yet well established. The reliability of BC ASSR thresholds to estimate hearing thresholds at 500, 1,000, 2,000, and 4,000 Hz is evaluated.

**Methods:**

A systematic search was conducted to identify studies involving normal-hearing (NH) and hearing-impaired (HI) participants of all ages. Outcomes were (1) the difference between ASSR behavioral and ASSR thresholds, and (2) ASSR thresholds. The risk of bias was evaluated using the Newcastle-Ottawa Scale. The certainty of the evidence was assessed using GRADE approach.

**Results:**

Twelve records met the inclusion criteria, yielding a total of 29 studies. Sample sizes ranged from 60 to 271 participants across frequencies and age groups. Record quality ranged from low to high. Data were synthesized using random-effects models due to heterogeneity. In NH adults, the mean differences between BC ASSR thresholds and behavioral thresholds were 17.0, 15.5, 13.4, and 12.1 dB at 500, 1,000, 2,000, and 4,000 Hz, respectively. In NH infants, mean BC ASSR thresholds were 17.2, 10.5, 26.1, and 19.9 dB HL at the same frequencies. In infants with conductive HL, BC ASSR threshold was 20.3 at 500 Hz. The certainty of the evidence was very low.

**Conclusions:**

Age and frequency impact BC ASSR thresholds, highlighting the need to develop correction values to accurately predict BC behavioral thresholds.

**Systematic review registration:** https://www.crd.york.ac.uk/PROSPERO/view/CRD42023422150.

## 1 Introduction

Auditory Steady-State Responses (ASSRs) are auditory evoked potentials that are used in clinical audiology to estimate hearing thresholds (Galambos et al., 1981; Lins and Picton, 1995; John et al., 1998, 2001b; Herdman and Stapells, 2001; Hall, 2015). Clinically, the technique is applicable for populations that are not able or not willing to provide behavioral thresholds, such as infants, children, and adults with intellectual difficulties or those with non-organic functional hearing loss.

The main advantage of the ASSR is that it allows objective simultaneous threshold measurements at several frequencies, reducing the testing time required (Picton et al., 2003). A second advantage is that interpretation is primarily objective, as it relies on statistical analysis of the signal in the frequency domain (Rance et al., 1995; Valdes et al., 1997; Luts and Wouters, 2005), whereas interpretation of Auditory Brainstem Response (ABR) is dependent primarily on subjective interpretation of the waveform in the time domain (Picton et al., 2003; Hall, 2015). The ASSR method is limited, in that, as the response is analyzed in the frequency domain, it does not provide information regarding neural integrity, and so cannot diagnose auditory neuropathy (Emara and Gabr, 2010). For this reason, the UK clinical ASSR protocol recommends ABR testing as the starting point for neonatal assessments (British Society of Audiology, 2023). For a comprehensive discussion of the strengths and weaknesses of ASSR, please refer to Rance (2008), Korczak et al. (2012), and Hall (2015).

Bone conduction (BC) testing is important to differentiate between conductive and sensorineural hearing loss, which in turn informs decisions about the urgency and type of intervention. Some information about conductive hearing loss can also be obtained through the click-ABR (Gorga et al., 1985), as well as tympanometry and acoustic immittance (Prieve et al., 2013a,b). In mixed hearing losses, BC levels inform the gain set on the hearing aids and thus ensure there is no under- or over-amplification (Dillon, 2012). This is particularly important for infants, where early intervention is crucial and obtaining outcome measures for hearing aid fitting is most difficult. Indeed, the need to test BC has been advocated by the Joint Committee on Infant Hearing (Joint Committee on Infant Hearing, 2007).

In infants, the skull is thinner, less ossified, and separated by open sutures and fontanelles, resulting in lower acoustic impedance and more efficient transmission of low-frequency BC. With maturation, increasing skull thickness and stiffness alter these transmission properties, providing a physiological basis for expected age-related differences in BC ASSR thresholds (Small and Stapells, 2008a).

Although there has been a substantial body of research on air conduction (AC) ASSRs (Korczak et al., 2012), a relatively smaller number of studies have focused on BC ASSR testing, especially when considering normative data for threshold estimation. In general, considerable heterogeneity exists in methods among ASSR studies (Tlumak et al., 2007; Korczak et al., 2012) and no universal testing protocol has been established (Michel and Jørgensen, 2017; Sininger et al., 2018; British Society of Audiology, 2023). To our knowledge, no previous meta-analysis has specifically synthesized BC ASSR thresholds at the four classic audiometric frequencies to evaluate their reliability for threshold estimation.

The objective of the meta-analysis was to assess whether hearing thresholds estimated by BC ASSR are reliable at the four classic audiometric frequencies (i.e., 500, 1,000, 2,000, and 4,000 Hz). The present study sought to synthesize the BC ASSR thresholds from studies conducted to date. Although our starting intention was to collect data from all ages, data from infants are very limited with many studies demonstrating methodological issues, such as different procedures to record BC ASSR (Small and Stapells, 2005; Small et al., 2007), lack of behavioral BC thresholds for comparison and frequency-specific hearing screening (Small and Stapells, 2008a).

## 2 Methods

The study was pre-registered in PROSPERO (CRD42023422150) and followed the PRISMA 2020 statement (Page et al., 2021a,b).

### 2.1 Literature search, data collection, and risk of bias

#### 2.1.1 Eligibility criteria

Eligibility criteria for study characteristics were based on the Population, Interventions, Comparators and Outcomes (PICO) framework (McKenzie et al., 2024). Population: both normal-hearing (NH) and hearing-impaired (HI) participants of all ages. Interventions: BC ASSR thresholds. Comparators: BC ASSR thresholds vs. BC behavioral thresholds of all ages, and in NH and HI participants. Outcomes: the primary outcome was the difference between BC behavioral and ASSR thresholds, while the secondary outcome was the BC ASSR thresholds. Additional co-factors were participants’ age and hearing threshold, testing levels and frequencies.

Only studies that reported BC ASSR thresholds were included. Studies were excluded if they met any of the following criteria: (1) not published in English, (2) pre-prints (i.e., not peer-reviewed), (3) reviews, (4) animal studies, (5) BC ASSR data collected from participants with a genetic syndrome or condition, (6) BC ASSR thresholds not reported, (7) BC ASSRs recorded in cochlear implant or active middle ear implant users, (8) BC ASSRs evoked by a 40 Hz modulation rate, or (9) unavailable as a full-text version. Studies involving genetic syndromes or conditions were excluded, as BC thresholds may be influenced by these conditions, thereby confounding the aim of establishing their correction values. BC ASSRs evoked by a 40 Hz modulation rate were excluded because ASSRs evoked by an 80 Hz modulation rate are recommended for assessing hearing thresholds (British Society of Audiology, 2023), and have different neural generators and amplitude than those evoked by a 40 Hz modulation rate (Rance, 2008; Luke et al., 2017; Weisz and Lithari, 2017). No restrictions were applied regarding the date of publication.

#### 2.1.2 Information sources and search strategy

The MEDLINE, Embase, and Cochrane Central Register of Controlled Trials (CENTRAL) databases were searched by the authors from May 2023 to August 2023 (see Supplementary Table 1 for the full search strategies). Using the exact same strategy, a further search was conducted in December 2025 to update (and verify) the initial search. Additionally, backward and forward citation searchings were performed using the Citationchaser Shiny app (Haddaway et al., 2022a). Specifically, all references of the included reports (backward search) and all studies that cited the most recent included report (forward search) were scanned. A systematic gray literature search was not performed.

#### 2.1.3 Selection process

The authors independently screened titles and abstracts of potentially eligible studies. Then, they independently reviewed full-text articles for inclusion. In both stages, disagreements between authors were solved by discussion.

#### 2.1.4 Data collection process

EP extrapolated data items and entered them into a spreadsheet. CG checked data accuracy. Disagreement was solved by re-checking articles. Data collection was performed by entering values from tables in the source articles, by synthesis of values from figures in the source articles via WebPlotDigitizer (Rohatgi, 2022; i.e., Fig 3 of Small and Stapells, 2008a and Fig 3 of Small and Hu, 2011), or by a table from the related dissertation (i.e., data of Casey and Small, 2014 were taken from Casey, 2012). We did not contact the corresponding authors to request any missing data.

#### 2.1.5 Data items

Data and information collected from the studies were the following: study (authors, year, and country), participants (sample size, age, and hearing threshold and group), equipment (device, bone conductor type and position, electrode montage), protocol (stimuli and their calibration, artifact rejection, average type, detection algorithm, noise criterion, and testing frequencies and levels). Missing data, such as participant age, are identified and reported in the Results section.

#### 2.1.6 Study risk of bias assessment

The Newcastle-Ottawa Scale (NOS) for cohort studies (Wells et al., 2018) was used to assess the risk of bias in the included studies. The assessment covered three categories (Selection, Comparability, and Outcome) and collectively identified over eight items. Items in the Selection and Outcome categories could be awarded a maximum of one star each, while the item in the Comparability category could receive up to two stars. Studies with 0–3 stars were considered high risk, those with 4–6 stars intermediate risk, and those with 7–9 stars low risk (Lo et al., 2014). As stated in the pre-registration, a minimum of three papers with at least three stars on the NOS tool was required for inclusion in the synthesis. No sensitivity analyses were planned. The two authors independently assessed the studies and resolved any discrepancies through discussion.

The NOS is designed for cohort studies with longitudinal outcomes, which are uncommon in audiological electrophysiology studies. In the literature, other tools are available, such as ROBINS-I (Risk Of Bias In Non-Randomized Studies of Interventions; Sterne et al., 2016), which is generally recommended (Sterne et al., 2024). However, NOS has similar reliability to ROBINS-I, while being less time-consuming and less complex (Zhang et al., 2021).

### 2.2 Effect measures, synthesis methods, and certainty assessment

#### 2.2.1 Effect measures

Analyses were performed on the BC ASSR thresholds, and on the difference between their behavioral and ASSR BC thresholds. The thresholds were analyzed as a function of participants’ age and testing frequency. Mean, SD, effect size and 95% confidence intervals (CIs) were calculated. The effect size, expressed in dB HL, was calculated both as the mean difference between BC ASSR and BC behavioral thresholds, and as the mean BC ASSR thresholds.

#### 2.2.2 Synthesis methods

The scope of the synthesis was twofold. First, it aimed to compare BC behavioral and ASSR thresholds for both NH and HI participants, with the goal of establishing correction values for accurately predicting BC behavioral thresholds from BC ASSR thresholds. Second, it involved pooling BC ASSR thresholds for NH participants to establish typical BC ASSR thresholds with the aim of using these as clinical discharge criteria.

However, only four studies (one in adults and three in infants) reported results for HI participants. In general, most articles reported only ASSR BC thresholds, i.e., behavioral thresholds were missing. Given the thresholds variability associated with age and frequency, data subgroups were made based on participants’ age (i.e., adults or infants) and on frequency (i.e., 500, 1,000, 2,000, and 4,000 Hz).

To assess statistical heterogeneity of the data, the I2 index (Deeks et al., 2024) was performed within each subgroup. Specifically, the I2 index measures the proportion of total variability due to between-study heterogeneity (Rücker et al., 2008). Either fixed-effect modeling (in case of I2 < 50%; i.e., low heterogeneity) or random-effect modeling (otherwise) are used to synthesize the data. The I2 index is based on the Chi-squared test, with a statistically significant (i.e., between-study heterogeneity) *p*-value threshold of < 0.10 (Deeks et al., 2024). In the fixed-effect model, the heterogeneity is neglected and the difference among studies is assumed as being due to chance. Instead, in the random-effect model, the heterogeneity is incorporated.

The objective of the meta-analysis was to assess whether hearing thresholds estimated by BC ASSR are reliable at the four classic audiometric frequencies (i.e., 500, 1,000, 2,000, and 4,000 Hz) and for both adults and infants. To be considered a reliable threshold, the 95% confidence intervals around the mean of the synthesised data should be within 10 dB. An error within 10 dB reflects both the 10 dB presentation steps used for ASSR stimuli (Small and Stapells, 2005) and the test–retest repeatability of ASSR (D’haenens et al., 2008; Israelsson et al., 2015), and is considered clinically acceptable in individuals with NH (D’haenens et al., 2008).

All analyses were run in R (version 4.3.1; R Core Team, 2024) using the packages *PRISMA2020* (Haddaway et al., 2022b), meta (Balduzzi et al., 2019), and the *Tidyverse* family (Wickham et al., 2019).

#### 2.2.3 Certainty assessment

The GRADE approach was used to assess the certainty of the evidence. As the reports included in the meta-analysis were non-randomized studies, by definition, the body of evidence initially began with a low-certainty rating. The following criteria were considered for downgrading the certainty of evidence, where appropriate: unexplained heterogeneity or inconsistency of results, indirectness of evidence, imprecision of results, and a high probability of publication bias (Schünemann et al., 2024). The GRADEpro GDT software was used to prepare the Summary of Findings tables.

## 3 Results

### 3.1 Study selection

**Figure 1:**
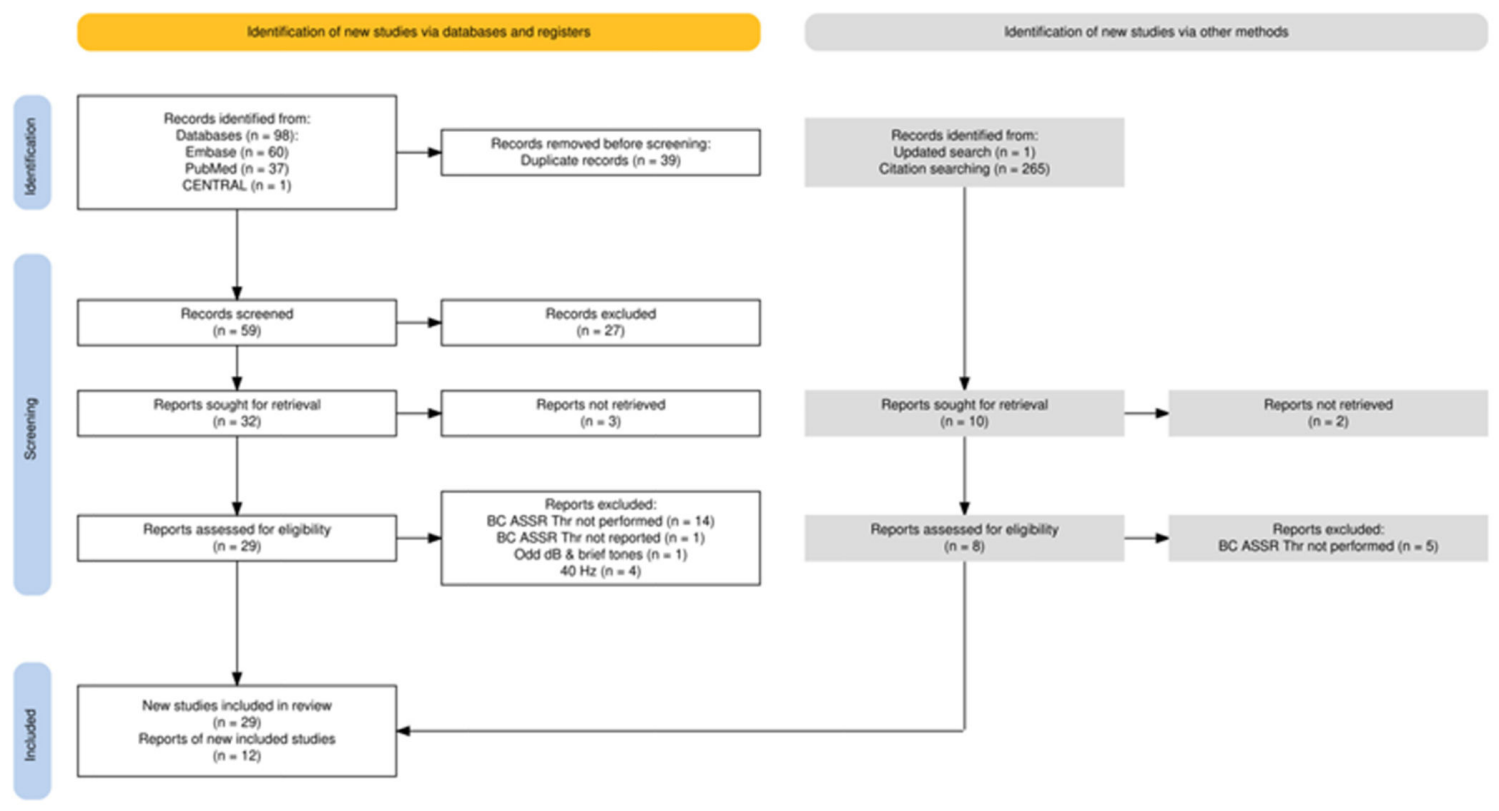
PRISMA 2020 flow diagram of the study selection procedure.

The database searches identified 98 records. After removal of duplicates, 59 records were screened. Twenty-seven records were excluded because they were not in English, were preprints or conference abstracts, were reviews, were not relevant, involved genetic conditions, or involved participants with cochlear implants or active middle ear implants. Three records were not available. Consequently, 29 records underwent full-text review. Of these, 20 records were excluded because they did not performed BC ASSR thresholds, did not report BC ASSR thresholds, reported dB values that could not be converted, or were conducted at 40 Hz. Citation searching of the included records identified 265 additional records, which were scanned, and two met the final selection criteria. The list of reasons for exclusion is provided in Supplementary Table 2. After this work was available as a preprint in medRxiv, an updated search identified Valeriote and Small (2024), which had previously been excluded as a preprint but has since been published. Therefore, a total of 12 records remained, which included 29 individual studies (Lins et al., 1996; Small and Stapells, 2005, 2006, 2008a,b; Small et al., 2007; Swanepoel et al., 2008; Ishida et al., 2011; Small and Hu, 2011; Casey and Small, 2014; Ismaila et al., 2016; Valeriote and Small, 2024). A summary of the selection procedure is presented in Figure 1.

The number of studies was larger than the number of reports, as several reports tested different groups of participants. Specifically, Small and Stapells (2006) measured thresholds in both preterm and post-term infants. Small et al. (2007) reported three different experiments involving three different groups of infants. Small and Stapells (2008a) had three different age groups. Small and Stapells (2008b) and Casey and Small (2014) recorded thresholds in infants and adults. Swanepoel et al. (2008) measured thresholds in NH infants and in three groups of HI infants (conductive HL [CHL], mild-to-moderate and severe-to-profound sensorineural hearing loss [SNHL]). Ishida et al. (2011) tested two groups of NH adults and a group of HI adults. Small and Hu (2011) reported thresholds obtained in young infants, older infants, and adults. Ismaila et al. (2016) measured thresholds in NH infants and in two groups of HI infants (mild-to-moderate and severe-to-profound SNHL). Valeriote and Small (2024) measured thresholds in NH infants and infants with CHL.

### 3.2 Study characteristics

The characteristics of the 12 included reports are presented in Tables 1, 2. The corresponding authors on seven out of 12 reports were based at the University of British Columbia (Vancouver, Canada) as the group led by David R. Stapells and Susan A. Small have produced many contributions in the field (Small and Stapells, 2005, 2006, 2008a,b; Small et al., 2007; Small and Hu, 2011; Casey and Small, 2014). The other reports were based at the University of Toronto (Canada; Lins et al., 1996), University of Pretoria (South Africa; Swanepoel et al., 2008), Al-Azhr University (Cairo, Egypt; Ismaila et al., 2016), and Royal University Hospital (Saskatoon, Canada; Valeriote and Small, 2024) with Susan A. Small as co-author. All reports tested participants within different age groups (e.g., adults and infants) and/or different hearing groups (e.g., NH and HI).

To generate and record the responses, the Rotman MASTER system (John and Picton, 2000) I and II were used in nine out of 12 reports, and the GSI AUDERA (Grason-Stadler, Eden Prairie, Minnesota, USA) used twice (Swanepoel et al., 2008; Ismaila et al., 2016). It is worth noting that the Rotman MASTER systems are no longer commercially available, which may have implications for the generalisability of the correction factors to second-generation devices or stimuli. The B71 bone transducer (Radioear, New Eagle, Pennsylvania, USA) was used in all reports. It was positioned on the temporal bone in nine reports, on the mastoid in two reports (Swanepoel et al., 2008; Ismaila et al., 2016), and on the forehead in one report (Lins et al., 1996). It is worth noting that a newer BC transducer, the B81, is currently commercially available, which may have implications for both behavioral thresholds (Fröhlich et al., 2018) and the generalisability of the correction factors.

Different types of stimuli were used across reports. 100% amplitude modulated (AM) tones were used in two reports (Lins et al., 1996; Small and Stapells, 2005), with mixed modulated tones (100% AM and 10% to 25% FM) in another eight reports (Small and Stapells, 2006, 2008b,a; Small et al., 2007; Swanepoel et al., 2008; Ishida et al., 2011; Small and Hu, 2011; Ismaila et al., 2016). Exponentially AM tones (100% AM and 5% FM) were used two reports (Casey and Small, 2014; Valeriote and Small, 2024). Carrier frequencies tested were 500, 1,000, 2,000, and 4,000 Hz in all studies, with 250 Hz also tested by Swanepoel et al. (2008). The ASSR stimuli were calibrated in dB HL in all studies except Lins et al. (1996), which used “air-equivalent SPL”. Online artifact rejection was set by all but two reports (Swanepoel et al., 2008; Ismaila et al., 2016), with threshold for rejection of epochs between ±40 and 80 μV. Weighted (John et al., 2001a), rather than linear averaging of sweeps was used in six reports (Small et al., 2007; Small and Stapells, 2008a,b; Ishida et al., 2011; Small and Hu, 2011; Casey and Small, 2014).

#### 3.2.1 Detection algorithm and noise criterion

Multiple detection algorithms have been employed for ASSR analysis: F ratio, Hotelling T2 (HT2), phase coherence squared (PC2), as well as combinations of the above (Valdes et al., 1997; Cebulla et al., 2001). The F ratio for hidden periodicity compares the power of the signal (i.e., the modulation frequency) to the mean power of N adjacent frequencies. The F ratio follows an F-distribution with [2 and N] degrees of freedom (Valdes et al., 1997; Picton et al., 2003). HT2 is considered repeated measurements of the variance of the response (Picton et al., 2003), as it is based on individual epochs rather than an average epoch (Valdes et al., 1997). HT2 is the two-dimensional analog of univariate *t*-tests, where the real and imaginary components of the epoch spectra form a two-dimensional vector. The HT2 test assesses whether the means of the real and imaginary components are significantly different from zero (Valdes et al., 1997; Picton et al., 2003). The PC2, in contrast, considers only the phase of the response and is based on the Rayleigh test (Cebulla et al., 2001; Picton et al., 2003).

An ASSR system requires an algorithm for determining the likelihood of the absence or presence of a response. For the former, usually a Signal to Noise Ratio (SNR) analysis and/or noise criterion threshold are involved and, for the latter, a Residual Noise (RN) analysis is required. The efficiency with which this is carried out will determine the accuracy in threshold estimation. It has been shown that increased number of sweeps will increase SNR and reduce RN resulting in increased threshold estimation accuracy (Luts and Wouters, 2004). It is therefore important to consider the effects of detection technique, the effects of number of sweeps, and noise criterion for stopping averaging in the current analysis.

The detection algorithms employed depended on the device. The Rotman MASTER I and II decision is based on F ratio while the GSI AUDERA is based on PC2 (see Luts and Wouters, 2005 for a comparison). In nine reports, the noise criterion for considering a response absent was defined as either noise < 11 nV and *p* > 0.05, or noise < 10 nV and *p* ≥ 0.30 (Small and Stapells, 2005, 2006, 2008a,b; Small et al., 2007; Ishida et al., 2011; Small and Hu, 2011; Casey and Small, 2014; Valeriote and Small, 2024). Other reports used a non-significant detection of ASSR for 64 sweeps (Lins et al., 1996), a noise criterion threshold of 1 μV (Swanepoel et al., 2008) or 1 mV^1^ (Ismaila et al., 2016).

#### 3.2.2 Effect measures

As stated in the pre-registration, a minimum of three papers with at least three stars on the NOS tool was required for inclusion in the synthesis. Since Lins et al. (1996) used a different calibration, their data were included only for comparisons between BC behavioral and BC ASSR thresholds, as these differences are independent of calibration.

Four reports, yielding five studies, compared BC behavioral and BC ASSR thresholds in NH adults (Lins et al., 1996; Small and Stapells, 2006; Ishida et al., 2011; Small and Hu, 2011). The data from these studies were used to carry out a meta-analysis.

BC ASSR thresholds in NH adults were estimated in six reports, yielding seven studies (Small and Stapells, 2005, 2008a,b; Ishida et al., 2011; Small and Hu, 2011; Casey and Small, 2014). The data from these studies were used to carry out a second meta-analysis.

Thresholds in HI adults were estimated only by Ishida et al. (2011), which was not sufficient to carry out a meta-analysis.

Only two studies (Casey and Small, 2014; Ismaila et al., 2016) compared BC behavioral and BC ASSR thresholds in NH infants. These data were not used for meta-analyses.

BC ASSR thresholds in NH infants were estimated in eight reports, yielding 13 studies (Small and Stapells, 2006, 2008a,b; Small et al., 2007; Swanepoel et al., 2008; Small and Hu, 2011; Casey and Small, 2014; Ismaila et al., 2016). The data from these studies were used to carry out a third meta-analysis.

BC ASSR thresholds in infants with CHL at 500 Hz were estimated in three reports (Swanepoel et al., 2008; Ismaila et al., 2016; Valeriote and Small, 2024). The data from these studies were used to carry out a fourth meta-analysis.

In total, four meta-analyses were performed: (1) BC ASSR thresholds vs. BC behavioral in NH adults, (2) BC ASSR thresholds in NH adults, (3) BC ASSR thresholds in NH infants, (4) BC ASSR thresholds in CHL infants at 500 Hz.

#### 3.2.3 Hearing threshold

For adult participants, behavioral AC and, in five studies, BC thresholds were obtained, so their hearing thresholds were clinically ascertained.

For infant participants, Casey and Small (2014) performed visual reinforcement audiometry to estimate hearing threshold at different frequencies (British Society of Audiology, 2014). Ismaila et al. (2016) performed play audiometry but reported only BC thresholds.

Hearing was screened via automatic auditory brain stem response (AABR) and/or distortion-product otoacoustic emissions (DPOAE) by five studies (Small and Stapells, 2006, 2008a,b; Small et al., 2007; Swanepoel et al., 2008) with also tympanograms by Swanepoel et al. (2008). Hearing screening in Small and Hu (2011) was based only on transient evoked otoacoustic emission (TEOAE). However, AABR and OAE do not provide frequency-specific estimates of auditory function. This is their main drawback for hearing screening (Dhar, 2012; Hall, 2015). Valeriote and Small (2024) performed AC and BC ABR at multiple frequencies, tympanograms and TEOAEs, which permit more accurate screening. The lack of frequency-specific hearing thresholds may have resulted in residual hearing loss at specific frequencies, which may have limited the validity of pooling NH infants.

#### 3.2.4 Same participants in multiple experiments or studies

Small et al. (2007) compared the effects of BC coupling methods (i.e., elastic-band vs. hand-held) and oscillator placements (temporal, mastoid and forehead) on ASSR threshold. The two coupling methods were recorded in the same infants; here we included the elastic-band thresholds because their standard deviation was smaller than for the hand-held thresholds. The three oscillator placements were recorded in the same infants; here we included temporal bone thresholds as it was the placement most commonly used in the other articles.

Two reports (Small and Stapells, 2008a; Small and Hu, 2011) combined new participants’ thresholds with those previously reported, or combined thresholds from different articles. These thresholds are included here because their mean and standard deviation were different.

### 3.3 Risk of bias in studies

A summary of these assessments is provided in Table 3. Six reports were classified as high risk (Small and Stapells, 2006; Small et al., 2007; Small and Stapells, 2008a,b; Swanepoel et al., 2008; Small and Hu, 2011) because the evaluation of hearing thresholds in infants was not frequency-specific (see above). Four reports were classified as intermediate risk (Lins et al., 1996; Small and Stapells, 2005; Casey and Small, 2014; Ismaila et al., 2016), and two study as low risk (Ishida et al., 2011; was Valeriote and Small, 2024). It is worth noting that Lins et al. (1996) had some unique features (i.e. six participants, placement of the bone conductor on the forehead rather than the temporal bone, and use of a different detection algorithm), which may place it at a somewhat higher risk of bias compared with other studies and current clinical practice. However, it was rated as intermediate risk.

Since none of the studies included follow-up, only one star was assigned in the Outcome category. Cumulatively, all studies received at least three stars and were therefore included in the synthesis.

### 3.4 Results of individual studies and syntheses

#### 3.4.1 BC ASSR vs. behavioral thresholds in NH adults

**Figure 2:**
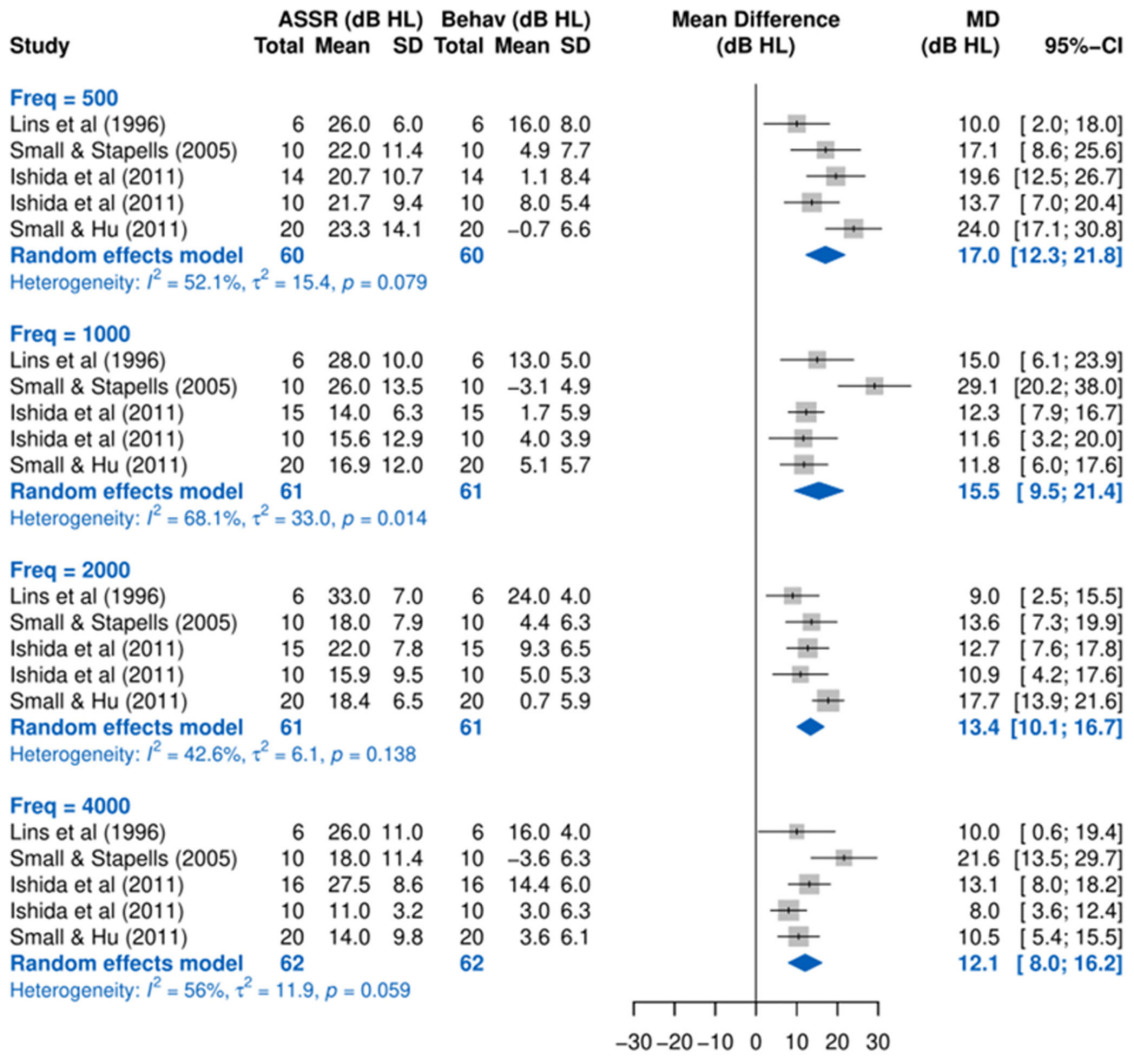
Forest plot for BC ASSR vs behavioral thresholds in NH adults as a function of the frequency. Behav: behavior; SD: standard deviation; MD: mean difference; CI: confident intervals.

Figure 2 shows the forest plot for BC ASSR vs. behavioral thresholds in NH adults as a function of the frequency (see also Supplementary Table 3). The number of participants was 60, 61, 61, and 62 at 500, 1,000, 2,000, and 4,000 Hz, respectively. This variation reflects differences in the reported thresholds in Ishida et al. (2011). Heterogeneity was less than 50% only at 2,000 Hz (I2 = 43%, *τ* 2 = 6.1, *p* = 0.14). It was high at 500 (I2 = 52%, *τ* 2 = 15.4, *p* = 0.08), 1,000 (I2 = 68%, *τ* 2 = 33.0, *p* = 0.01), and 4,000 Hz (I2 = 58%, *τ* 2 = 11.9, *p* = 0.06). For consistency, a random effects model was carried out for each frequency, using the inverse variance method, the restricted maximum-likelihood estimator for *τ* 2, and untransformed (raw) means. Means (and 95% confident intervals) of the random effects model for each frequency were 17.0 (12.3/21.8) dB at 500 Hz, 15.5 (9.5/21.4) dB at 1,000 Hz, 13.4 (10.1/16.7) dB at 2,000 Hz, and 12.1 (8.0/16.2) dB at 4,000 Hz.

#### 3.4.2 BC ASSR thresholds in NH adults

**Figure 3:**
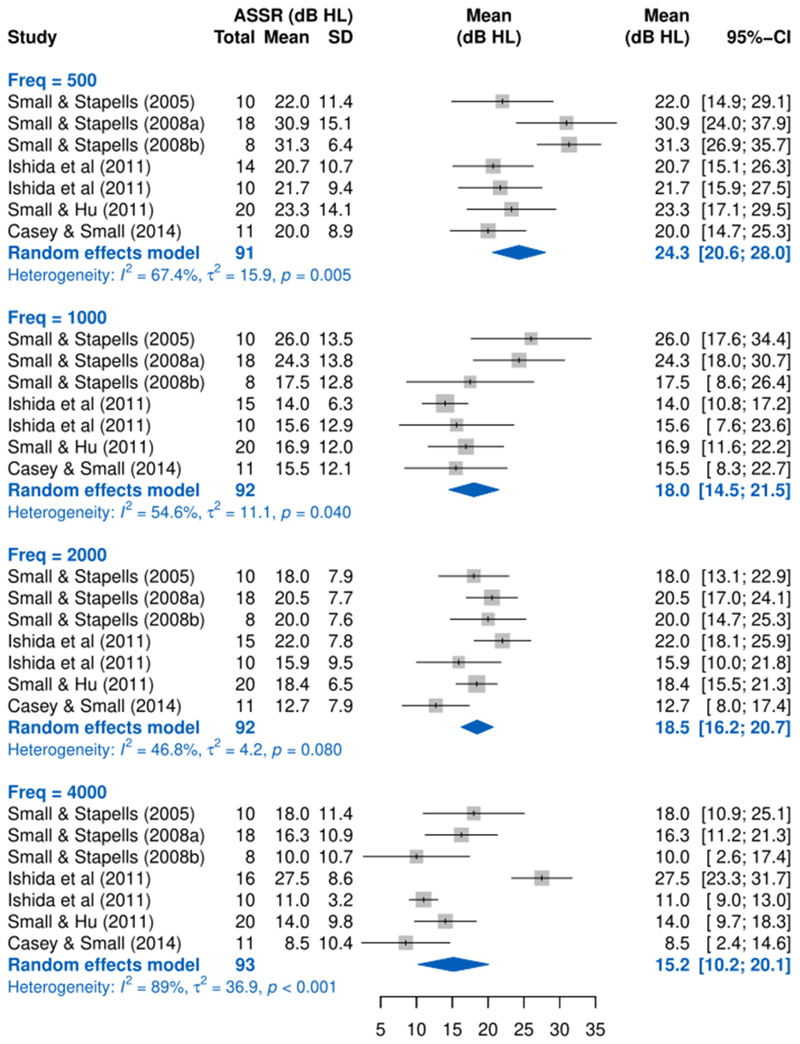
Forest plot for BC ASSR thresholds in NH adults as a function of the frequency. SD: standard deviation; CI: confident intervals.

Figure 3 shows the forest plot for BC ASSR thresholds in NH adults as a function of the frequency (see also Supplementary Table 4). Seven studies reported BC ASSR thresholds in NH adults, with Ishida et al. (2011) reporting the thresholds on two different cohorts. The number of participants per frequency was between 91 and 93. Heterogeneity was less than 50% only at 2,000 Hz (*I*^2^ = 47%, *τ* ^2^ = 4.2, *p* = 0.08). It was high at 500 (*I*^2^ = 67%, *τ* ^2^ = 15.9, *p* < 0.01), 1,000 (*I*^2^ = 55%, *τ* ^2^ = 11.1, *p* = 0.04), and 4,000 Hz (*I*^2^ = 89%, *τ* ^2^ = 36.9, *p* < 0.01). For consistency, a random effects model was carried out for each frequency, using the inverse variance method, the restricted maximum-likelihood estimator for *τ* ^2^, and untransformed (raw) means.

Means (and 95% confident intervals) of the random effects model for each frequency were 24.3 (20.6/28.0) dB HL at 500 Hz, 18.0 (14.5/21.5) dB HL at 1,000 Hz, 18.5 (16.2/20.7) dB HL at 2,000 Hz, and 15.2 (10.2/20.1) dB HL at 4,000 Hz.

#### 3.4.3 BC ASSR thresholds in NH infants

**Figure 4:**
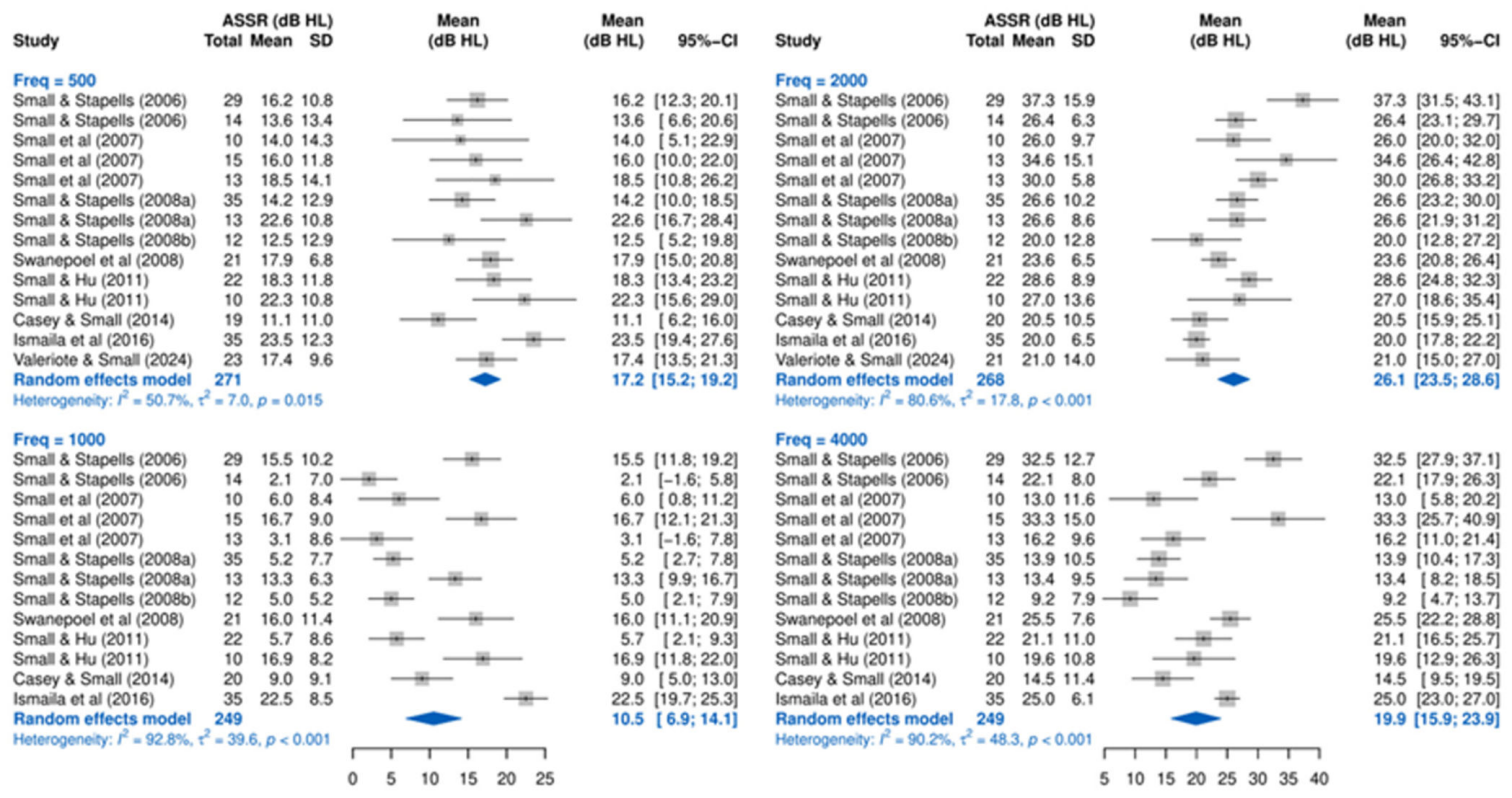
Forest plot for BC ASSR thresholds in NH infants as a function of the frequency. SD: standard deviation; CI: confident intervals.

Figure 4 shows the forest plot for BC ASSR thresholds in NH infants as a function of the frequency (see also Supplementary Table S5 and Table S6, as well as Table S7 for a sensitivity analysis). Seven reports reported BC ASSR thresholds in NH infants, with additional four reports reporting the thresholds on different cohorts (Small and Stapells, 2006, 2008a; Small et al., 2007; Small and Hu, 2011). The number of participants per frequency was between 249 and 271. High heterogeneity was present at each frequency as *I*^2^ = 51% (*τ* ^2^ = 7.0, *p* < 0.01) at 500 Hz; *I*^2^ = 93% (*τ* ^2^ = 39.6, *p* < 0.01) at 1,000 Hz; *I*^2^ = 81% (*τ* ^2^ = 18.0, *p* < 0.01) at 2,000 Hz; and *I*^2^ = 90% (*τ* ^2^ = 48.3, *p* < 0.01) at 4,000 Hz. Therefore, a random effects model was carried out, using the inverse variance method, a restricted maximum-likelihood estimator for *τ* ^2^ and untransformed (raw) means.

Means (and 95% confident intervals) of the random effects model for each frequency were 17.2 (15.2/19.2) dB HL at 500 Hz, 10.5 (6.9/14.1) dB HL at 1,000 Hz, 26.1 (23.5/28.6) dB HL at 2,000 Hz, and 19.9 (15.9/23.9) dB HL at 4,000 Hz.

#### 3.4.4 BC ASSR thresholds in CHL infants at 500 Hz

Three reports estimated BC ASSR thresholds in CHL infants at 500 Hz, for a total of 85 participants (see also Supplementary Table 8). High heterogeneity was present having *I*^2^ = 82% (*τ* ^2^ = 12.9, *p* < 0.01). Therefore, a random effects model was carried out, using the inverse variance method, a restricted maximum-likelihood estimator for *τ* ^2^ and untransformed (raw) means. Mean (and 95% confident intervals) of the random effects model was 20.3 (15.6/24.9) dB HL.

#### 3.4.5 Spurious responses

Of particular interest are the minimum intensity levels at which spurious (artifactual) results were observed. Supplementary Table 9 summarizes the spurious results reported in the included studies. The level at which spurious responses occur increased as a function of frequency. Similarly, the percentage of participants with spurious responses increased with frequency. Therefore, the BC ASSR threshold at low frequencies, such as 500 Hz, and moderate intensities, such as 40 dB HL, may not be reliable. Swanepoel et al. (2008) questioned the clinical value of BC ASSR thresholds measured at or below 500 Hz. Current UK guidance recommends that BC ASSR should not be performed at 500 Hz, and beyond 40 dB nHL for frequencies between 1,000 and 4,000 Hz (British Society of Audiology, 2023).

BC stimuli presented at moderate and high intensities elicited spurious responses, primarily at 500 Hz and 1,000 Hz. At these frequencies and range of intensities, BC ASSR amplitudes were abnormally larger when compared either to those at low intensities or to AC ASSR amplitudes (Picton and John, 2004). Importantly, these spurious responses could lead to an underestimation of thresholds. The reasons for these findings, along with potential solutions, are discussed elsewhere (Picton and John, 2004; Small and Stapells, 2004, 2005).

### 3.5 Certainty assessment

The GRADE Summary of Findings tables for BC ASSR thresholds vs. behavioral thresholds in NH adults are presented in Table 4, those for BC ASSR thresholds in NH adults and infants are presented in Table 5, and those for BC ASSR in CHL infant in Table 6. As the included studies were cohort studies, the body of evidence began with a low-certainty rating. The certainty was subsequently downgraded to very low due to serious inconsistency, reflected by high heterogeneity (in both adults and infants), and serious indirectness (in infants), as the evaluation of hearing thresholds was not frequency-specific.

## 4 Discussion

The present meta-analysis included 12 records spanning 28 years of research in BC ASSR. To our knowledge, no previous meta-analyses have specifically considered BC ASSR (but see Small and Stapells, 2017). Only 12 records have been published over three decades (1996–2026), highlighting the limited research in this area, despite BC thresholds being essential for differentiating conductive from sensorineural hearing loss, and, hence, for informing decisions on the type of intervention.

An accurate BC ASSR threshold estimation relies on precise and reliable correction values that allow the prediction of behavioral thresholds from the measured ASSRs values. A possible definition of a reliable BC ASSR threshold is that its 95% confidence interval is within 10 dB of the mean of each model. The width of the confidence interval ranged from 2.2 to 6.0 dB HL, indicating that it remained within 10 dB.

Our main finding is the elevation of BC ASSR thresholds, as a function of both age group and frequency. For NH adults, the difference between behavioral and ASSR thresholds ranged from 12.1 to 17.0 dB (i.e., the first meta-analysis § 4.4.1), while BC ASSR thresholds ranged from 15.2 to 24.3 dB HL across the frequency range of 500-4,000 Hz (i.e., the second meta-analysis § 4.4.1). For NH infants, BC ASSR thresholds ranged from 10.5 to 26.4 dB HL within the same frequency range.

For infants with conductive hearing loss, ASSR thresholds are elevated by 20 dB HL at 500 Hz. The latter is particularly important, as an elevated AC threshold with a normal BC threshold indicates the presence of conductive loss, whereas an elevated BC threshold indicates the presence of sensory loss (Small and Stapells, 2017). However, given the risk of spurious responses at 500 Hz, the synthesized mean should be considered provisional.

The BSA Early Assessment Guidance (British Society of Audiology, 2021, Appendix H) provides provisional corrections for ABR thresholds as a function of frequency and age. For NH adults, the provisional corrections range from 11.8 to 20.4 dB, which are similar to our findings. However, ABR is a different electrophysiological technique with different neural generators, waveform morphology, and threshold estimation procedures, provisional correction factors for ABR thresholds cannot be applied to BC ASSR in clinical practice.

To explore a less-well established technique such as BC ASSRs, thresholds should be compared with a gold standard technique such as behavioral thresholds. This is achievable in adults, but not possible in neonates as behavioral assessment only becomes possible around 7 months and even then, a lengthy period may be required to determine accurate behavioral thresholds. A compromise would be to compare the BC ASSR thresholds to those obtained using BC ABR.

The certainty of the evidence, evaluated using the GRADE approach, was very low for all four meta-analyses, as they were based solely on non-randomized studies, for which the starting rating is, by definition, low. Planning a randomized study in the field of audiological electrophysiology is challenging, particularly in infants, and even more so in those with hearing loss. A theoretical randomized study could involve one group of infants undergoing BC ASSR and another undergoing BC ABR; however, this would be unethical because the correction factors for BC ASSR have not yet been established. Instead, in both clinical practice and research, the general objective is to collect as much data as possible from infants. BC ASSR may represent a viable method for estimating BC thresholds, pending the establishment and validation of appropriate correction values.

### 4.1 Heterogeneity

Heterogeneity was strongly apparent across the studies. This was due to multiple factors, such as stimuli, statistical indices, stopping criteria, and threshold definitions used. Furthermore, several studies reported a large standard deviation (up to 16.9 dB HL) of BC ASSR thresholds. Nevertheless, the average BC ASSR thresholds were consistently positive, meaning they were always higher than the behavioral thresholds. The high level of heterogeneity among studies may reduce the clinical validity of the correction factors.

Because of the small number of studies available, it was not possible to assess the effect of recording parameters on the BC ASSR thresholds. A previous meta-analysis on AC ASSR thresholds revealed that the effect of recording parameters, such as type of modulation (i.e., amplitude or mixed modulated) and maximum number of sweeps, varied for different frequencies and hearing status (Tlumak et al., 2007).

### 4.2 Infant thresholds

The measure of BC ASSR thresholds for infants (less than 1 year) and children (more than 1 year old) should be interpreted with extra caution because of their age. Specifically, the reported age varied from 34.5 weeks’ post-conceptional age (PCA) to 4.5 years. Including such a large age range might be a confound for BC ASSR thresholds as, at least for AC ASSR thresholds, thresholds tend to decrease in NH infants as a function of age (Rance and Tomlin, 2006; Sousa et al., 2017). Our rationale for including the full age range was that the heterogeneity and variability of the studies were greater than any potential effects of age. For instance, ASSR threshold (and standard deviation) at 2,000 Hz was nearly independent of Age: 20.0 (12.8) dB HL in 21-week-old infants (Small and Stapells, 2008b), 20.50 (10.5) dB HL in 10.5-month infants (Casey and Small, 2014), 23.6 (6.5) dB HL in 3.6-year-old children (Swanepoel et al., 2008), and 20.0 (6.5) dB HL in 4.5-year-old children (Ismaila et al., 2016). However, to rule out the possible confound due to Age, a further meta-analysis was conducted excluding the studies in children (Swanepoel et al., 2008; Ismaila et al., 2016). Means (and 95% confident intervals) of the random effects model for each frequency were 16.4 (14.5/18.3) dB HL at 500 Hz, 8.9 (5.6/12.1) dB HL at 1,000 Hz, 27.0 (24.3/29.7) dB HL at 2,000 Hz, and 18.9 (14.3/23.4) dB HL at 4,000 Hz (see also Supplementary Table 7). Thus, the overall mean difference was 0.65 dB HL between the model including children and the one excluding them.

It was not possible to perform a sensitivity analysis for BC ASSR thresholds in CHL, as only three studies were included; therefore, these results should be considered preliminary and not used as a clinical reference.

A further issue with the infant thresholds is that hearing screening was not frequency-specific, which may have limited the validity of pooling NH infants and may partly explain the elevation at 2,000 Hz.

### 4.3 BC ASSR thresholds for adults and infants

**Figure 5:**
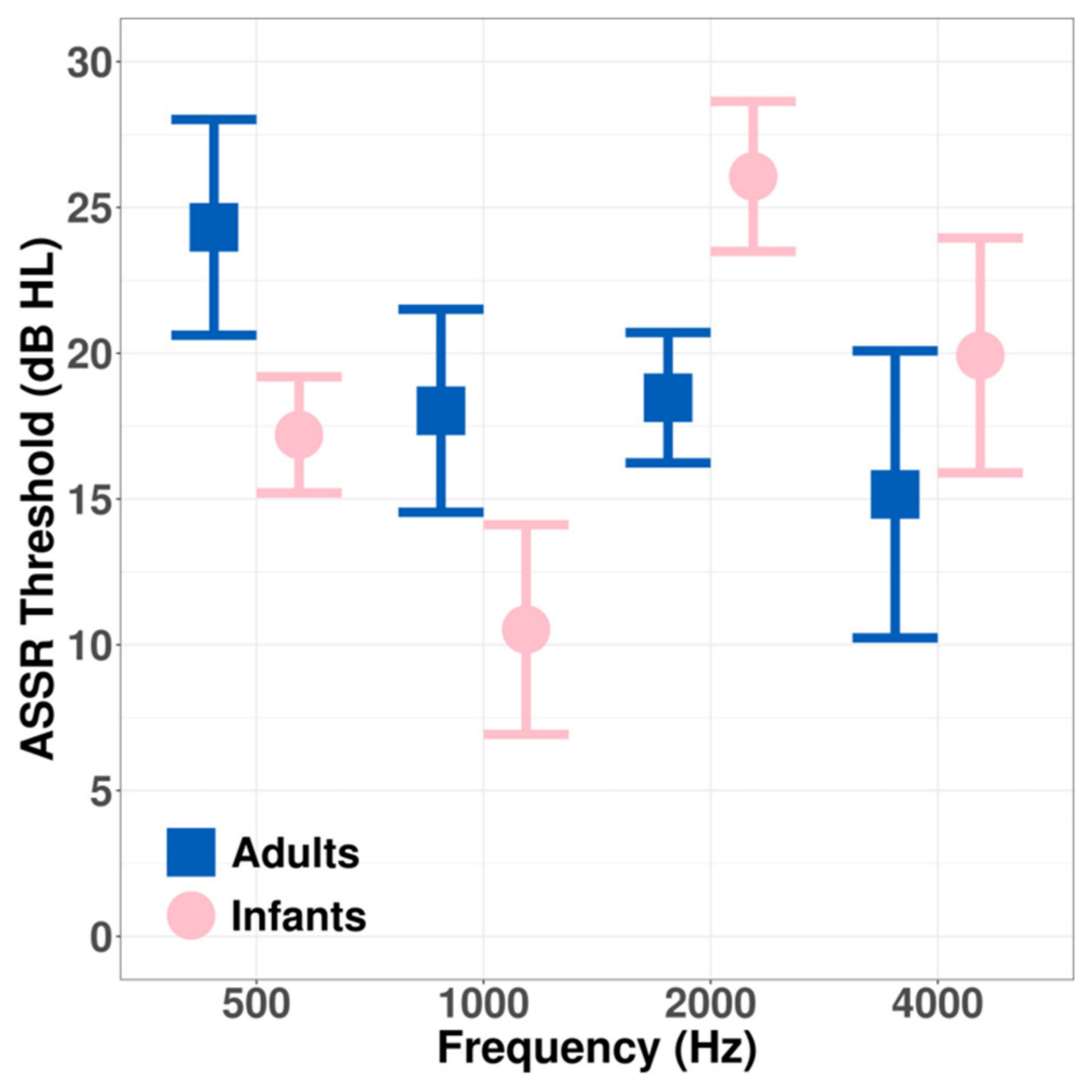
Summary of the meta-analysis results for BC ASSR thresholds in adults (beige square) and infants (purple circle). The ASSR threshold dB (attenuator) as a function of the frequency.

Among others, Casey and Small (2014) compared BC ASSR thresholds in adults and infants. Figure 5 shows a summary of the meta-analysis results for BC ASSR thresholds in adults (blue squares) and infants (pink circles) in the same conditions, i.e., only ASSR.

Adult thresholds are higher at low frequencies (500 and 1,000 Hz) and lower at high frequencies (2,000 and 4,000 Hz) when compared to those of infants. The adult threshold at 2,000 Hz was about the same as those at 1,000 and 4,000 Hz. Instead, the infant threshold at 2,000 Hz was clearly higher than those at other frequencies. Interestingly, adult thresholds at 2,000 Hz showed significantly less heterogeneity than those in infants, which may be explained by differences in middle-ear ossicle inertia, as this may play a role in bone conduction at 2,000 Hz (Stenfelt, 2011). BC stimuli in infants are effectively more intense than the same stimuli in adults, likely due to differences in skull maturation (Small and Stapells, 2008a). Since BC ASSR thresholds change as a function of age and frequency, correction values are needed for different population and frequency.

### 4.4 Variability of BC ASSR thresholds

In the current meta-analyses, the standard deviations for BC ASSR thresholds across frequency was between 10.91 (at 2,000 Hz) and 24.3 (at 4,000 Hz) dB HL for adults, and between 16.3 (at 500 Hz) and 32.4 (at 4,000 Hz) dB HL for infants. Comparing these with AC ASSR thresholds, the standard deviation was reported between 9.9 and 12.1 dB HL for adults (Tlumak et al., 2007), and between 7 and 15 dB SL for infants (Table 9-2 in Rance, 2008). Comparing BC ASSR with BC tone burst ABR, the standard deviation was between 6.2 and 10.5 dB HL for adults, and between 7.2 and 10 for infants (Foxe and Stapells, 1993; Vander Werff et al., 2009). Therefore, the inter-subject variability across frequency in BC ASSR threshold was larger than those in AC ASSR and BC ABR thresholds. However, these comparisons are illustrative rather than direct, and the larger BC ASSR standard deviations may partly reflect heterogeneous recording protocols across the included studies, rather than an inherent property of the BC ASSR technique itself.

It has been suggested that the substantial variability in BC ASSR thresholds may be due to factors such as variations in transducer placement, the condition of occluded vs. non-occluded ears, differences in stimulus calibration methods (Small and Stapells, 2005). Other possible contributing factors include test duration, artifact rejection thresholds, average type, detection algorithms, and noise criteria.

The aforementioned standard deviation values refer to inter-subject variability. Since BC ASSR thresholds can be influenced by several factors, intra-subject variability should also be evaluated. Future studies should assess the test-retest reliability of BC ASSR threshold (Israelsson et al., 2015).

### 4.5 Possible limitations

Our aim was to synthesize BC ASSR thresholds at the four standard audiometric frequencies. In doing so, we encountered several limitations.

Beside the range of age of the infants discussed earlier (§5.2), another limitation was that most of the included studies were conducted by Stapells and Small (§4.2). Contribution from other laboratories or countries should help to improve the quality of research such as, for instance, removing risk of bias. Therefore, most of the included studies originated from the same research group, which may reflect shared methodological preferences, equipment calibration, and participant recruitment practices, as well as a potential risk of data duplication, leading to systematic bias. Completely independent datasets would have been ideal. A related issue was that most of the thresholds were collected using the Rotman MASTER system (Table 2), a first-generation system that is no longer available. A key limitation is the complete absence of BC ASSR normative data from a second generation system (Sininger et al., 2018), which limits clinical applicability and should be addressed in future research.

The majority of the reports were classified as high risk, with only two considered low risk (§4.3). The high risk of bias across publications is a limitation of this research field. A further limitation is the lack of HI thresholds (§4.2.2) required to conduct a meta-analysis in populations where correction factors are needed.

There were also limitations in the review processes used. For instance, the exclusion of non-English articles, conference abstracts, and gray literature may have restricted data diversification and, potentially, the quality of the research (§3.1.1). Nevertheless, we are confident in our outcome and the overall conclusions.

## 6 Conclusions

Findings from this meta-analysis suggest that ASSR can be a reliable technique to estimate BC thresholds in NH populations, particularity once correction values have been established. In the frequency range of 500–4,000 Hz, the ASSR threshold ranged from 15 to 24 dB HL in NH adults, and from 10 to 26 dB HL in NH infants. Conductive HL leads to an elevation of 20 dB HL at 500 Hz. Although based on very low-certainty evidence and requiring prospective clinical validation, provisional BC ASSR correction values for NH adults involve subtracting approximately 17, 15.5, 13.4, and 12.1 dB from BC ASSR thresholds at 500, 1,000, 2,000, and 4,000 Hz, respectively. Future work is needed to validate and refine BC ASSR correction values in NH infants, as well as to start investigating both HI adults and infants. Only then can we reliably define normative BC ASSR thresholds for different populations.

### Registration

This systematic review has been registered in the international prospective register of systematic reviews (PROSPERO) under the registration number: CRD42023422150.

## Supplementary Material

**Supplementary material**

The Supplementary Material for this article can be found online at: https://www.frontiersin.org/articles/10.3389/fauot.2026.1837322/full#supplementary-material

Supplementary Material

## Figures and Tables

**Table 1:** Study characteristics of the 12 included studies.

Study	Country	Sample	Age (mean)	Hearing status	Hearing estimation
Lins et al. (1996)	Canada	8 adults	NR	NH	Behavioral AC & BC @ 500 to 4000 Hz
Small and Stapells (2005)	Canada	10 adults	Range: 18-48 years	NH	Behavioral AC&BC ≤ 20 dB HL @ 250 to 8000 Hz
Small and Stapells (2006)	Canada	29 preterm infants	34.5 week PCA	NH	AABR at 35 dB nHL
		14 postterm infants	17 week	NH	DPOAE
Small et al. (2007)	Canada	10 postterm infants	17 week	NH	AABR at 35 dB nHL or DPOAE
		15 preterm infants (9were included in the analysis of ASSR threshold)	34.5 week PCA	NH	AABR at 35 dB nHL
		13 postterm infants	15 week	NH	DPOAE
Small and Stapells (2008a)	Canada	35 young infants (but 9 from Small et al., 2007 and Small and Stapells, 2008b); 14 from Small and Stapells, 2006); 12 from Small and Stapells, 2008b^2^)	16 week	NH	AABR at 35 dB nHL or DPOAE
		13 older infants	18.2 months	NH	
		18 adults (but 10 from Small and Stapells, 2005)	22.9 years	NH	Behavioral AC & BC thresholds ≤ 25 dB HL @ 500-4000 Hz
Small and Stapells (2008b)	Canada	14 infants (with 1 participated also in Small et al., 2007)	21 week	NH	DPOAE
		11 adults	23 years	NH	Behavioral AC thresholds ≤ 25 dB HL @ 500-4000 Hz
Swanepoel et al. (2008)	South Africa	13 infants (21 ears)	3.6 years	NH	Tympanograms, DPOAE, AC click-evoked ABR thresholds, AC ASSR thresholds
		18 infants (35 ears)	2.7 years	Conductive HL	
		7 infants (13 ears)	2.5 years	Mild-to-moderate SNHL	
		13 infants (23 ears)	2.2 years	Severe-to-profound SNHL	
Ishida et al. (2011)	Canada	16 adults	26.8 years	NH	Behavioral AC thresholds ≤ 20 dB HL @ 500-4000 Hz
		10 adults	24.6 years	NH	
		40 adults	46.0 years	SNHL	Behavioral AC thresholds > 20& ≤ 55dB HL @ 500-4000Hz; & BC thresholds
Small and Hu (2011)	Canada	young infants (but for 12 from Small et al., 2007)	2.1 months	NH	TEOAE
		10 older infants	15.3 months	NH	
		20 adults	26.5 years	NH	Behavioral AC & BC thresholds ≤ 25 dB HL @ 500-4000 Hz
Casey and Small (2014)	Canada	23 infants	10.5 months	NH	TEOAE or tympanometry
		12 adults	29.9 years	NH	Behavioral AC & BC thresholds ≤ 25 dB HL @ 500-4000 Hz & air-bone gaps ≤10 dB
Ismaila et al. (2016)	Egypt	20 infants (35 ears)	4.5 years	NH	AC play audiometry mean = 15 dB HL, AC click-evoked ABR thresholds mean = 30 dBnHL, tympanograms
		20 infants (36 ears)		Mild-to-moderate SNHL	AC &BC play audiometry mean = 30-50 dB HL, AC click-evoked ABR thresholds mean = 40-60 dBnHL, tympanograms
		20 infants (35 ears)		Conductive HL	AC play audiometry mean = 25-50 dB HL, Normal BC play audiometry, AC click-evoked ABR elevated, tympanograms
Valeriote and Small (2024)	Canada	Up to 23 ears	7.36 weeks	NH	AC &BC ABR, TEOAE &tympanograms
		15 ear	6.71 weeks	Conductive HL	AC &BC ABR, TEOAE &tympanograms

AABR: automatic auditory brain stem response; AC: air-conduction thresholds; BC: bone-conduction thresholds; DPOAE: distortion-product otoacoustic emissions; HI: hearing-impaired participants; NH: normal-hearing participants; NR: not reported: PCA: post-conceptional age; SNHL: sensorineural hearing loss; TEOAE: transient evoked otoacoustic emission.

**Table 2:** Hearing test information of the 12 included studies.

Study	Equipment	Placement	Stimulus	Sweep	Electrode montage	Artifact rejection	Average	Detection algorithm (CR)	Noise criterion (RA)	Testing levels
Lins et al. (1996)	NR	Forehead	Modulated tones with 100% AM Multiple CF: 0.5, 1, 2, & 4 kHz MF: 77, 85, 93, & 101 Hz	16 epochs of 512 data points and lasted a total of 12.06 seconds.	Cz and at the nape f the neck	Epoch > ±40 uV in amplitude rejected.	Normal	F ratio & HT^2^ with *p* < 0.05	64 sweeps were not SIG	Started at 50 dB SPL, decreased in 10 dB step until all resp were absent
Small and Stapells (2005)	Rotman MASTER research system	Temporal Bone	Modulated tones with 100% AM Multiple CF: 0.5, 1, 2, & 4 kHz MF: 77, 85, 93, & 101 Hz	16 epochs of 1,024 data points and lasted a total of 13.107 s.	Non-inverting on Cz. Inverting at the nape of the neck, just below the hairline. Ground on high forehead.	Epoch > ±40 uV in amplitude rejected.	Normal	F ratio with *p* < 0.05	noise < 11 nV &*p* > 0.05 OR noise < 10 nV & *p* ≥ 0.30	10 dB steps at: (1) 50 to 10dB HL for non-inverted stimuli, (2) 50 to 30 dB HL for inverted stimuli, and (3) 50 to 20 dB HL for alternated stimuli.
Small and Stapells (2006)	Rotman MASTER research system	Temporal Bone	Modulated tones with 100% AM & 25% FM Multiple CF: 0.5, 1,2,& 4 kHz MF: 77, 85, 93, & 101 Hz	16 epochs of 1,024 data points and lasted a total of 13.107 s. A minimum of 7 sweeps per condition.	Non-inverting on midline at the high forehead. Inverting at the nape of the neck, just below the hairline. Ground at the low forehead.	Epoch > ±40 uV in amplitude rejected.	Normal	F ratio with *p* < 0.05 for at least two consecutive sweeps.	noise < 11 nV &*p* > 0.05 OR noise < 10 nV &*p*≥ 0.30	
Small et al. (2007)	Rotman MASTER research system	Temporal Bone	Modulated tones with 100% AM & 25% FM Multiple CF: 0.5, 1,2,& 4 kHz MF: 77, 85, 93, & 101 Hz	16 epochs of 1,024 data points and lasted a total of 13.107 s. A minimum of 7 sweeps per condition.	Non-inverting on forehead. Inverting at the nape of the neck, just below the hairline. Ground on high forehead.	Epoch > ±40 uV in amplitude rejected.	Weighted	F ratio with *p* < 0.05 for at least two consecutive sweeps.	noise < 11 nV &*p* > 0.05 OR noise < 10 nV &*p*≥ 0.30	10-dB steps from 50 to − 10dB HL
Small and Stapells (2008a)	Rotman MASTER research system	Temporal Bone	Modulated tones with 100% AM & 25% FM Multiple CF: 0.5, 1,2,& 4kHzMF: 77, 85, 93, & 101 Hz	16 epochs of 1,024 data points and lasted a total of 13.107 s. A minimum of 7 sweeps per condition.	For 23 of the 35 young infants, all older infants and adults: Non-inverting on forehead. Inverting at the nape of the neck. Ground on high forehead. For 12 of the 35 young infants, 2 2 inverting on the L & R mastoids	Epoch > ±40 uV in amplitude rejected.	Weighted	F ratio with *p* < 0.05 for at least two consecutive sweeps.	noise < 11 nV &*p* > 0.05 OR noise < 10 nV & *p* ≥ 0.30	10-dB steps
Small and Stapells (2008b)	Rotman MASTER research system	Temporal Bone	Modulated tones with 100% AM & 25% FM Multiple CF: 0.5, 1,2,& 4 kHz MF: 77, 85, 93, & 101 Hz	16 epochs of 1,024 data points and lasted a total of 13.107 s. A minimum of 7 sweeps per condition.	Non-inverting on high forehead. Inverting on mastoids. Ground on low forehead.	Epoch > ±40 uV in amplitude rejected.	Weighted	F ratio with *p* < 0.05 for at least two consecutive sweeps.	noise < 11 nV &*p* > 0.05 OR noise < 10 nV & *p* ≥ 0.30	10-dB steps from 50 to − 10dB HL
Swanepoel et al. (2008)	GSI AUDERA	Mastoid	Modulated tones with 100% AM & 10% FM Multiple CF: 0.25, 0.5, 1,2,& 4kHzMF: 67, 74, 81, 88, &95 Hz	NA	Non-inverting on high forehead (Fz). Inverting on ipsilateral mastoid. Ground on contralateral mastoid.			PC^2^ with *p* <0.03	1 uV	10-& 5-dB steps
Ishida et al. (2011)	Rotman MultiMASTER	Temporal Bone	Modulated tones with 100% AM & 25% FM Multiple CF: 0.5, 1,2,& 4 kHz MF: 77, 85, 93, & 101 Hz	16 epochs of 1,024 data points and lasted a total of 13.107 s. A min of 12 sweeps per condition.	Non-inverting on Cz. Inverting on midline at the nape. Ground on high forehead.	Epoch > ±57 uV in amplitude rejected.	Weighted	F ratio with *p* < 0.05 for at least 7 consecutive sweeps.	Noise1 < 11 nV &*p* > 0.05 OR ASSR amp < 10nV& *p* ≥ 0.30	Started at 20 or 40 dB HL, increased or decreased in 10-dB steps
Small and Hu (2011)	Rotman MultiMASTER	Temporal Bone	Modulated tones with 100% AM & 25% FM Multiple CF: 0.5, 1,2,& 4kHzMF: 77, 85, 93, & 101 Hz	16 epochs of 1,024 data points and lasted a total of 13.107 s. A min of 10 sweeps per condition.	Non-inverting on high forehead. Inverting on ipsilateral mastoids. Ground on forehead.	Epoch > ±80 uV in amplitude rejected.	Weighted	F ratio with *p* < 0.05 for at least 2 consecutive sweeps.	noise < 11 nV &*p* > 0.05 OR ASSR amp < 10 nV & *p* ≥ 0.30	Random start between 10 and 30 dB HL, increased or decreased in 10-dB steps
Casey and Small (2014)	Rotman MultiMASTER	Temporal Bone	Exponential modulated tones with 100% AM& 5% FM Multiple CF: 0.5, 1,2,& 4 kHz MF: 78, 85, 93, & 101 Hz	16 epochs of 1,024 data points and lasted a total of 13.107 s. A min of 10 consecutive sweeps	Non-inverting on Fz. Inverting on ipsilateral mastoids. Ground on low forehead.	Epoch > ±80 uV in amplitude rejected.	Weighted	F ratio with *p* < 0.05 for at least 3 consecutive sweeps.	noise < 11 nV &*p* > 0.05 OR ASSR amp < 10 nV & *p* ≥ 0.30	Random start between 10 and 30 dB HL, increased or decreased in 10-dB steps
Ismaila et al. (2016)	Gsi AUDERA	Mastoid	Modulated tones with 100% AM & 10% FM Multiple CF: 0.5, 1,2,& 4kHz MF: 74, 81, 88, & 95 Hz	NA	Non-inverting on high forehead (Fz). Inverting on ipsilateral mastoid. Ground on contralateral mastoid.			PC^2^ with *p* <0.03	1 mV^3^	10-dB steps
Valeriote and Small (2024)	MASTER II	Mastoid	Exponential modulated tones with 100% AM Multiple CF: 0.5, 1, 2, & 4 kHz MF: 78, 85, 93, & 101 Hz	16 epochs of 1,024 data points and lasted a total of 13.11 s. A min of 10 consecutive sweeps	Non-inverting on Vertex. Inverting on ipsilateral mastoids. Ground on low forehead.			F ratio with *p* < 0.05 for at least 3 consecutive sweeps.	noise < 15 nV &*p* > 0.05	Started at 30 dB HL, increased 20-dB or decreased in 10-dB steps

AM: amplitude modulation; CR: clear response; CF: carrier frequency; FM: frequency modulation; HL: hearing loss; HT2: Hotelling T2; MF: modulation frequency; NR: not reported; PC2: phase coherence squared; RA: response absent; SNHL: sensorineural hearing loss.

**Table 3:** Risk of bias for each of the included study.

Study	Selection (out of 4)	Comparability (out of 2)	Outcome (out of 3)	Total (out of 9)	Category
Lins et al. (1996)	2	1	1	4	Intermediate
Small and Stapells (2005)	3	0	1	4	Intermediate
Small and Stapells (2006)	1	1	1	3	High
Small et al. (2007)	1	1	1	3	High
Small and Stapells (2008a)	1	1	1	3	High
Small and Stapells (2008b)	1	1	1	3	High
Swanepoel et al. (2008)	1	1	1	3	High
Ishida et al. (2011)	4	1	1	6	Low
Small and Hu (2011)	1	1	1	3	High
Casey and Small (2014)	2	1	1	4	Intermediate
Ismaila et al. (2016)	2	1	1	4	Intermediate
Valeriote and Small (2024)	4	1	1	6	Low

**Table 4:** Summary of Findings tables for BC ASSR thresholds vs. behavioral thresholds in NH adults.

BC ASSR thresholds compared to Behavioral BC thresholds in NH adults			
Patient or population: NH adults Setting: Audiology departments Intervention: BC ASSR thresholds Comparison: BC behavioral thresholds			
Outcomes	No of participants (studies)	Certainty of the evidence (GRADE)	Anticipated absolute effects BC ASSR thresholds vs. Behav BC thresholds
			BC ASSR thresholds vs. Behav BC thresholds
500 Hz	60 (5 non-randomized studies)	⊕◯◯◯ Very low^1^	MD 17.0 **dB HL higher** (12.3 higher to 21.8 higher)
1,000 Hz	61 (5 non-randomized studies)	⊕◯◯◯ Very low^1^	MD 15.5 **dB HL higher** (9.5 higher to 21.4 higher)
2,000 Hz	61 (5 non-randomized studies)	⊕◯◯◯ Very low^1^	MD 13.4 **dB HL higher** (10.1 higher to 16.7 higher)
4,000 Hz	62 (5 non-randomized studies)	⊕◯◯◯ Very low^1^	MD 12.1 **dB HL higher** (8.0 higher to 16.2 higher)

MD, mean difference; Behav, Behavioral. **GRADEWorking Group grades of evidence** **High certainty:** we are very confident that the true effect lies close to that of the estimate of the effect. **Moderate certainty:** we are moderately confident in the effect estimate: the true effect is likely to be close to the estimate of the effect, but there is a possibility that it is substantially different. **Low certainty:** our confidence in the effect estimate is limited: the true effect may be substantially different from the estimate of the effect. **Very low certainty:** we have very little confidence in the effect estimate: the true effect is likely to be substantially different from the estimate of effect. **Explanations** ^1^ High heterogeneity.

**Table 5:** Summary of Findings tables for BC ASSR thresholds in NH adults and infants.

BC ASSR thresholds in NH adults and infants			
Patient or population: NH adults and infants Setting: Audiology departments Intervention: BC ASSR thresholds			
Outcomes	No of participants (studies)	Certainty of the evidence (GRADE)	Anticipated absolute effects
			BC ASSR thresholds
Adults			
500 Hz	97 (8 non-randomized studies)	⊕◯◯◯ Very low^1^	Mean **24.6 dB HL higher** (21.4 higher to 27.8 higher)
1,000 Hz	98 (8 non-randomized studies)	⊕◯◯◯ Very low^1^	mean **19.2 dB HL higher** (15.5 higher to 23.0 higher)
2,000 Hz	98 (8 non-randomized studies	⊕◯◯◯ Very low^1^	Mean **20.0 dB HL higher** (16.1 higher to 23.9 higher)
4,000 Hz	99 (8 non-randomized studies)	⊕◯◯◯ Very low^1^	Mean **16.3 dB HL higher** (11.4 higher to 21.1 higher)
Infants			
500 Hz	271 (14 non-randomized studies)	⊕◯◯◯ Very low^1,2^	mean **17.2 dB HL higher** (15.0 higher to 19.4 higher)
1,000 Hz	249 (13 non-randomized studies)	⊕◯◯◯ Very low^1,2^	mean **10.5 dB HL higher** (6.9 higher to 14.1 higher)
2,000 Hz	268 (14 non-randomized studies)	⊕◯◯◯ Very low^1,2^	Mean **26.4 dB HL higher** (23.7 higher to 29.1 higher)
4,000 Hz	249 (13 non-randomized studies)	⊕◯◯◯ Very low^1,2^	mean **19.9 dB HL higher** (15.9 higher to 23.9 higher)

**GRADEWorking Group grades of evidence** **High certainty:** we are very confident that the true effect lies close to that of the estimate of the effect. **Moderate certainty:** we are moderately confident in the effect estimate: the true effect is likely to be close to the estimate of the effect, but there is a possibility that it is substantially different. **Low certainty:** our confidence in the effect estimate is limited: the true effect may be substantially different from the estimate of the effect. **Very low certainty:** we have very little confidence in the effect estimate: the true effect is likely to be substantially different from the estimate of effect. **Explanations**

1 High heterogeneity.

2 Evaluation of hearing thresholds was not frequency-specific.

**Table 6:** Summary of findings tables for BC ASSR thresholds in CHL infants.

BC ASSR thresholds in CHL infants			
Patient or population: CHL infants Setting: Audiology departments Intervention: BC ASSR thresholds			
Outcomes	No of participants (studies)	Certainty of the evidence (GRADE)	Anticipated absolute effects
			BC ASSR thresholds
500 Hz	85 (3 non-randomized studies)	⊕◯◯◯ Very low^1,2^	mean 20.3 **dB HL higher** (15.6 higher to 24.9 higher)

**GRADEWorking Group grades of evidence** **High certainty:** we are very confident that the true effect lies close to that of the estimate of the effect. **Moderate certainty:** we are moderately confident in the effect estimate: the true effect is likely to be close to the estimate of the effect, but there is a possibility that it is substantially different. **Low certainty:** our confidence in the effect estimate is limited: the true effect may be substantially different from the estimate of the effect. **Very low certainty:** we have very little confidence in the effect estimate: the true effect is likely to be substantially different from the estimate of effect. **Explanations**

1 High heterogeneity

2 Evaluation of hearing thresholds was not frequency-specific.

## Data Availability

The original contributions presented in the study are included in the article/Supplementary material, further inquiries can be directed to the corresponding author.

## Footnotes

1 The manuscript states that ‘a noise criterion level of 1mV was used’. This value is extremely high and not physiologically realistic; it is likely a typographical error, and the intended value was 1 μV, as typically used in GSI Audera equipment (e.g., Swanepoel et al., 2008).

## Abbreviations

AC: air-conduction threshold
AMEI: active middle ear implant
AM: amplitude modulation
ASSR: auditory steady-state response
BC: bone-conduction threshold
CHL: conductive hearing loss
CT2: circular T2 test
HI: hearing-impaired participant
DPOAE: distortion-product otoacoustic emissions
HT2: hotelling T2
NH: normal-hearing participant
MSC: magnitude squared coherence
NOS: Newcastle-Ottawa Scale
PC2: phase coherence squared
PCA: post-conceptional age
PICO: population, xinterventions, comparators and outcomes
SNHL: sensorineural hearing loss
TEOAE: transient evoked otoacoustic emission
