# Supplementary material for: A meta-analysis of bone conduction 80 Hz auditory steady-state response thresholds in adults and infants
Source: Front Audiol Otol. Author manuscript; Available in PMC 2026 Aug 3. (PMC7619303; doi:10.3389/fauot.2026.1837322)
Supplement: Supplementary Material [file EMS217166-supplement-Supplementary_Material.pdf]

A meta-analysis of bone conduction 80 Hz auditory-steady state response thresholds in adults & infants

Emanuele Perugia & Constantina Georga

Supplementary Material

Contents

A Study selection **2**

B Syntheses of adult thresholds **4**

C Syntheses of infant thresholds **6**

D Spurious (non-auditory) responses **10**

E Data **11**

List of Tables

S1 Search strategy . . . 2
S2 Characteristics of excluded studies table . . . 3
S3 BC ASSR vs behavioural thresholds in NH adults as a function of the frequency. . . . 4
S4 BC ASSR thresholds in NH adults as a function of the frequency. . . . 5
S5 BC ASSR thresholds in NH infants at 500 and 1000 Hz . . . 6
S6 BC ASSR thresholds in NH infants at 2000 and 4000 Hz . . . 7
S7 BC ASSR thresholds in NH infants, excluding studies in children (sensitivity analysis) . . 8
S8 BC ASSR thresholds in CHL infants at 500 Hz. . . . 9
S9 Spurious (non-auditory) responses. . . . 10
S10 Data . . . 15

A Study selection

Database	Search details
PubMed	("Bone Conduction" OR "Bone-Conducted" OR "Bone Vibrator") AND ("ASSR" OR "Auditory Steady-State Response" OR "EFR" OR "Envelope Following Response")
Cochrane Library	#1 MeSH descriptor: [Bone Conduction] explode all trees<br>#2 (Bone Conduction):ti,ab,kw<br>#3 (Bone-Conducted):ti,ab,kw<br>#4 (Bone Vibrator):ti,ab,kw<br>#5 #1 OR #2 OR #3 OR #4<br>#6 (Auditory Steady-State Response):ti,ab,kw<br>#7 (ASSR):ti,ab,kw<br>#8 (Envelope Following Response):ti,ab,kw<br>#9 (EFR):ti,ab,kw<br>#10 #6 OR #7 OR #8 OR #9<br>#11 #5 AND #10
Embase	(("Bone Conduction" OR "Bone-Conducted" OR "Bone Vibrator") AND ("ASSR" OR "Auditory Steady-State Response" OR "EFR" OR "Envelope Following Response")).mp. [mp=title, abstract, heading word, drug trade name, original title, device manufacturer, drug manufacturer, device trade name, keyword heading word, floating subheading word, candidate term word]

Table S1: Search strategy

Study	Reason
Brennan et al. (2010)	ASSR BC Thr Not Done
Brooke et al. (2009)	ASSR BC Thr Not Done
Çelik et al. (2016)	ASSR BC Thr Not Done
Cone-Wesson et al. (2002)	ASSR BC Thr Not Done
D'haenens et al. (2009)	ASSR BC Thr Not Done
Dabbous et al. (2017)	40 Hz
Dabbous et al. (2019)	40 Hz
de Bressieux et al. (2025)	ASSR BC Thr Not Done
DeJonckere et al. (2021)	40 Hz
Dimitrijevic et al. (2002)	ASSR BC Thr Not Done
Guerrero-Aranda et al. (2016)	BC ASSR No Data
Hansen and Small (2012)	ASSR BC Thr Not Done
Komazec et al. (2010)	40 Hz
Michel and Jørgensen (2017)	ASSR BC Thr Not Done
Mo et al. (2011)	Odd dB & Brief Tones
Small and Stapells (2004)	ASSR BC Thr Not Done
Small et al. (2014)	ASSR BC Thr Not Done
Small and Love (2014)	ASSR BC Thr Not Done
Small and Hansen (2012)	ASSR BC Thr Not Done
Sininger et al. (2018)	ASSR BC Thr Not Done
Tarawneh et al. (2022)	ASSR BC Thr Not Done
Torres-Fortuny et al. (2016)	ASSR BC Thr Not Done
Van Maanen and Stapells (2010)	ASSR BC Thr Not Done
Wang et al. (2020)	ASSR BC Thr Not Done
Watkin et al. (2011)	ASSR BC Thr Not Done

Table S2: Characteristics of excluded studies table

B Syntheses of adult thresholds

Study	ASSR (dB HL)			Behavioural (dB HL)			MD (dB HL)	95%-CI
	Total	Mean	SD	Total	Mean	SD		
**Freq = 500 Hz**								
Lins et al. (1996)	6	26.0	6.0	6	16.0	8.0	10.0	[2.0; 18.0]
Small and Stapells (2005)	10	22.0	11.4	10	4.9	7.7	17.1	[8.6; 25.6]
Ishida et al. (2011)	14	20.7	10.7	14	1.1	8.4	19.6	[12.5; 26.7]
Ishida et al. (2011)	10	21.7	9.4	10	8.0	5.4	13.7	[7.0; 20.4]
Small and Hu (2011)	20	23.3	14.1	20	-0.7	6.6	24.0	[17.1; 30.8]
**Random Effect model**	**60**			**60**			17.0	**[12.3; 21.8]**
**Freq = 1000 Hz**								
Lins et al. (1996)	6	28.0	10.0	6	13.0	5.0	15.0	[6.1; 23.9]
Small and Stapells (2005)	10	26.0	13.5	10	-3.1	4.9	29.1	[20.2; 38.0]
Ishida et al. (2011)	15	14.0	6.3	15	1.7	5.9	12.3	[7.9; 16.7]
Ishida et al. (2011)	10	15.6	12.9	10	4.0	3.9	11.6	[3.2; 20.0]
Small and Hu (2011)	20	16.9	12.0	20	5.1	5.7	11.8	[6.0; 17.6]
**Random Effect model**	**61**			**61**			15.5	**[9.5; 21.4]**
**Freq = 2000 Hz**								
Lins et al. (1996)	6	33.0	7.0	6	24.0	4.0	9.0	[2.5; 15.5]
Small and Stapells (2005)	10	18.0	7.9	10	4.4	6.3	13.6	[7.3; 19.9]
Ishida et al. (2011)	15	22.0	7.8	15	9.3	6.5	12.7	[7.6; 17.8]
Ishida et al. (2011)	10	15.9	9.5	10	5.0	5.3	10.9	[4.2; 17.6]
Small and Hu (2011)	20	18.4	6.5	20	0.7	5.9	17.7	[13.9; 21.6]
**Random Effect model**	**61**			**61**			13.4	**[10.1; 16.7]**
**Freq = 4000 Hz**								
Lins et al. (1996)	6	26.0	11.0	6	16.0	4.0	10.0	[0.6; 19.4]
Small and Stapells (2005)	10	18.0	11.4	10	-3.6	6.3	21.6	[13.5; 29.7]
Ishida et al. (2011)	16	27.5	8.6	16	14.4	6.0	13.1	[8.0; 18.2]
Ishida et al. (2011)	10	11.0	3.2	10	3.0	6.3	8.0	[3.6; 12.4]
Small and Hu (2011)	20	14.0	9.8	20	3.6	6.1	10.5	[5.4; 15.5]
**Random Effect model**	**62**			**62**			12.1	**[8.0; 16.2]**

Table S3: BC ASSR vs behavioural thresholds in NH adults as a function of the frequency. SD: standard deviation; MD: mean difference; CI: confident intervals.

Study	ASSR (dB HL) Total	Mean	SD	Mean (dB HL)	95%-CI
**Freq = 500 Hz**					
Small and Stapells (2005)	10	22.0	11.4	22.0	[14.9; 29.1]
Small and Stapells (2008a)	18	30.9	15.1	30.9	[24.0; 37.9]
Small and Stapells (2008b)	8	31.3	6.4	31.3	[26.9; 35.7]
Ishida et al. (2011)	14	20.7	10.7	20.7	[15.1; 26.3]
Ishida et al. (2011)	10	21.7	9.4	21.7	[15.9; 27.5]
Small and Hu (2011)	20	23.3	14.1	23.3	[17.1; 29.5]
Casey and Small (2014)	11	20.0	8.9	20.0	[14.7; 25.3]
**Random Effect model**	**91**			**24.3**	**[20.6; 28.0]**
**Freq = 1000 Hz**					
Small and Stapells (2005)	10	26.0	13.5	26.0	[17.6; 34.4]
Small and Stapells (2008a)	18	24.3	13.8	24.3	[18.0; 30.7]
Small and Stapells (2008b)	8	17.5	12.8	17.5	[8.6; 26.4]
Ishida et al. (2011)	15	14.0	6.3	14.0	[10.8; 17.2]
Ishida et al. (2011)	10	15.6	12.9	15.6	[7.6; 23.6]
Small and Hu (2011)	20	16.9	12.0	16.9	[11.6; 22.2]
Casey and Small (2014)	11	15.5	12.1	15.5	[8.3; 22.7]
**Random Effect model**	**92**			**18.0**	**[14.5; 21.5]**
**Freq = 2000 Hz**					
Small and Stapells (2005)	10	18.0	7.9	18.0	[13.1; 22.9]
Small and Stapells (2008a)	18	20.5	7.7	20.5	[17.0; 24.1]
Small and Stapells (2008b)	8	20.0	7.6	20.0	[14.7; 25.3]
Ishida et al. (2011)	15	22.0	7.8	22.0	[18.1; 25.9]
Ishida et al. (2011)	10	15.9	9.5	15.9	[10.0; 21.8]
Small and Hu (2011)	20	18.4	6.5	18.4	[15.5; 21.3]
Casey and Small (2014)	11	12.7	7.9	12.7	[8.0; 17.4]
**Random Effect model**	**92**			**18.5**	**[16.2; 20.7]**
**Freq = 4000 Hz**					
Small and Stapells (2005)	10	18.0	11.4	18.0	[10.9; 25.1]
Small and Stapells (2008a)	18	16.3	10.9	16.3	[11.2; 21.3]
Small and Stapells (2008b)	8	10.0	10.7	10.0	[2.6; 17.4]
Ishida et al. (2011)	16	27.5	8.6	27.5	[23.3; 31.7]
Ishida et al. (2011)	10	11.0	3.2	11.0	[9.0; 13.0]
Small and Hu (2011)	20	14.0	9.8	14.0	[9.7; 18.3]
Casey and Small (2014)	11	8.5	10.4	8.5	[2.4; 14.6]
**Random Effect model**	**93**			**15.2**	**[10.2; 20.1]**

Table S4: BC ASSR thresholds in NH adults as a function of the frequency. SD: standard deviation; CI: confident intervals.

C Syntheses of infant thresholds

Study	ASSR (dB HL) Total	Mean	SD	Mean (dB HL)	95%-CI
**Freq = 500 Hz**					
Small and Stapells (2006)	29	16.2	10.8	16.2	[12.3; 20.1]
Small and Stapells (2006)	14	13.6	13.4	13.6	[6.6; 20.6]
Small et al. (2007)	10	14.0	14.3	14.0	[5.1; 22.9]
Small et al. (2007)	15	16.0	11.8	16.0	[10.0; 22.0]
Small et al. (2007)	13	18.5	14.1	18.5	[10.8; 26.2]
Small and Stapells (2008a)	35	14.2	12.9	14.2	[10.0; 18.5]
Small and Stapells (2008a)	13	22.6	10.8	22.6	[16.7; 28.4]
Small and Stapells (2008b)	12	12.5	12.9	12.5	[5.2; 19.8]
Swanepoel et al. (2008)	21	17.9	6.8	17.9	[15.0; 20.8]
Small and Hu (2011)	22	18.3	11.8	18.3	[13.4; 23.2]
Small and Hu (2011)	10	22.3	10.8	22.3	[15.6; 29.0]
Casey and Small (2014)	19	11.1	11.0	11.1	[6.2; 16.0]
Ismaila et al. (2016)	35	23.5	12.3	23.5	[19.4; 27.6]
Valeriote and Small (2024)	23	17.4	9.6	17.4	[13.5; 21.3]
**Random Effect model**	**271**			**17.2**	**[15.2; 19.2]**
**Freq = 1000 Hz**					
Small and Stapells (2006)	29	15.5	10.2	15.5	[11.8; 19.2]
Small and Stapells (2006)	14	2.1	7.0	2.1	[-1.6; 5.8]
Small et al. (2007)	10	6.0	8.4	6.0	[0.8; 11.2]
Small et al. (2007)	15	16.7	9.0	16.7	[12.1; 21.3]
Small et al. (2007)	13	3.1	8.6	3.1	[-1.6; 7.8]
Small and Stapells (2008a)	35	5.2	7.7	5.2	[2.7; 7.8]
Small and Stapells (2008a)	13	13.3	6.3	13.3	[9.9; 16.7]
Small and Stapells (2008b)	12	5.0	5.2	5.0	[2.1; 7.9]
Swanepoel et al. (2008)	21	16.0	11.4	16.0	[11.1; 20.9]
Small and Hu (2011)	22	5.7	8.6	5.7	[2.1; 9.3]
Small and Hu (2011)	10	16.9	8.2	16.9	[11.8; 22.0]
Casey and Small (2014)	20	9.0	9.1	9.0	[5.0; 13.0]
Ismaila et al. (2016)	35	22.5	8.5	22.5	[19.7; 25.3]
**Random Effect model**	**249**			**10.5**	**[6.9; 14.1]**

Table S5: BC ASSR thresholds in NH infants at 500 and 1000 Hz. SD: standard deviation; CI: confident intervals.

Study	ASSR (dB HL) Total	Mean	SD	Mean (dB HL)	95%-CI
**Freq = 2000 Hz**					
Small and Stapells (2006)	29	37.3	15.9	37.3	[31.5; 43.1]
Small and Stapells (2006)	14	26.4	6.3	26.4	[23.1; 29.7]
Small et al. (2007)	10	26.0	9.7	26.0	[20.0; 32.0]
Small et al. (2007)	13	34.6	15.1	34.6	[26.4; 42.8]
Small et al. (2007)	13	30.0	5.8	30.0	[26.8; 33.2]
Small and Stapells (2008a)	35	26.6	10.2	26.6	[23.2; 30.0]
Small and Stapells (2008a)	13	26.6	8.6	26.6	[21.9; 31.2]
Small and Stapells (2008b)	12	20.0	12.8	20.0	[12.8; 27.2]
Swanepoel et al. (2008)	21	23.6	6.5	23.6	[20.8; 26.4]
Small and Hu (2011)	22	28.6	8.9	28.6	[24.8; 32.3]
Small and Hu (2011)	10	27.0	13.6	27.0	[18.6; 35.4]
Casey and Small (2014)	20	20.5	10.5	20.5	[15.9; 25.1]
Ismaila et al. (2016)	35	20.0	6.5	20.0	[17.8; 22.2]
Valeriote and Small (2024)	21	21.0	14.0	21.0	[15.0; 27.0]
**Random Effect model**	**268**			**26.1**	**[23.5; 28.6]**
**Freq = 4000 Hz**					
Small and Stapells (2006)	29	32.5	12.7	32.5	[27.9; 37.1]
Small and Stapells (2006)	14	22.1	8.0	22.1	[17.9; 26.3]
Small et al. (2007)	10	13.0	11.6	13.0	[5.8; 20.2]
Small et al. (2007)	15	33.3	15.0	33.3	[25.7; 40.9]
Small et al. (2007)	13	16.2	9.6	16.2	[11.0; 21.4]
Small and Stapells (2008a)	35	13.9	10.5	13.9	[10.4; 17.3]
Small and Stapells (2008a)	13	13.4	9.5	13.4	[8.2; 18.5]
Small and Stapells (2008b)	12	9.2	7.9	9.2	[4.7; 13.7]
Swanepoel et al. (2008)	21	25.5	7.6	25.5	[22.2; 28.8]
Small and Hu (2011)	22	21.1	11.0	21.1	[16.5; 25.7]
Small and Hu (2011)	10	19.6	10.8	19.6	[12.9; 26.3]
Casey and Small (2014)	20	14.5	11.4	14.5	[9.5; 19.5]
Ismaila et al. (2016)	35	25.0	6.1	25.0	[23.0; 27.0]
**Random Effect model**	**249**			**19.9**	**[15.9; 23.9]**

Table S6: BC ASSR thresholds in NH infants at 2000 and 4000 Hz. SD: standard deviation; CI: confident intervals.

Study	ASSR (dB HL) Total	Mean	SD	Mean (dB HL)	95%-CI
**Freq = 500 Hz**					
Small and Stapells (2006)	29	16.2	10.8	16.2	[12.3; 20.1]
Small and Stapells (2006)	14	13.6	13.4	13.6	[6.6; 20.6]
Small et al. (2007)	10	14.0	14.3	14.0	[5.1; 22.9]
Small et al. (2007)	15	16.0	11.8	16.0	[10.0; 22.0]
Small et al. (2007)	13	18.5	14.1	18.5	[10.8; 26.2]
Small and Stapells (2008a)	35	14.2	12.9	14.2	[10.0; 18.5]
Small and Stapells (2008a)	13	22.6	10.8	22.6	[16.7; 28.4]
Small and Stapells (2008b)	12	12.5	12.9	12.5	[5.2; 19.8]
Small and Hu (2011)	22	18.3	11.8	18.3	[13.4; 23.2]
Small and Hu (2011)	10	22.3	10.8	22.3	[15.6; 29.0]
Casey and Small (2014)	19	11.1	11.0	11.1	[6.2; 16.0]
Valeriote and Small (2024)	23	17.4	9.6	17.4	[13.5; 21.3]
**Random Effect model**	**215**			**16.4**	**[14.5; 18.3]**
**Freq = 1000 Hz**					
Small and Stapells (2006)	29	15.5	10.2	15.5	[11.8; 19.2]
Small and Stapells (2006)	14	2.1	7.0	2.1	[-1.6; 5.8]
Small et al. (2007)	10	6.0	8.4	6.0	[0.8; 11.2]
Small et al. (2007)	15	16.7	9.0	16.7	[12.1; 21.3]
Small et al. (2007)	13	3.1	8.6	3.1	[-1.6; 7.8]
Small and Stapells (2008a)	35	5.2	7.7	5.2	[2.7; 7.8]
Small and Stapells (2008a)	13	13.3	6.3	13.3	[9.9; 16.7]
Small and Stapells (2008b)	12	5.0	5.2	5.0	[2.1; 7.9]
Small and Hu (2011)	22	5.7	8.6	5.7	[2.1; 9.3]
Small and Hu (2011)	10	16.9	8.2	16.9	[11.8; 22.0]
Casey and Small (2014)	20	9.0	9.1	9.0	[5.0; 13.0]
**Random Effect model**	**193**			**8.9**	**[5.6; 12.1]**
**Freq = 2000 Hz**					
Small and Stapells (2006)	29	37.3	15.9	37.3	[31.5; 43.1]
Small and Stapells (2006)	14	26.4	6.3	26.4	[23.1; 29.7]
Small et al. (2007)	10	26.0	9.7	26.0	[20.0; 32.0]
Small et al. (2007)	13	34.6	15.1	34.6	[26.4; 42.8]
Small et al. (2007)	13	30.0	5.8	30.0	[26.8; 33.2]
Small and Stapells (2008a)	35	26.6	10.2	26.6	[23.2; 30.0]
Small and Stapells (2008a)	13	26.6	8.6	26.6	[21.9; 31.2]
Small and Stapells (2008b)	12	20.0	12.8	20.0	[12.8; 27.2]
Small and Hu (2011)	22	28.6	8.9	28.6	[24.8; 32.3]
Small and Hu (2011)	10	27.0	13.6	27.0	[18.6; 35.4]
Casey and Small (2014)	20	20.5	10.5	20.5	[15.9; 25.1]
Valeriote and Small (2024)	21	21.0	14.0	21.0	[15.0; 27.0]
**Random Effect model**	**212**			**27.0**	**[24.3; 29.7]**
**Freq = 4000 Hz**					
Small and Stapells (2006)	29	32.5	12.7	32.5	[27.9; 37.1]
Small and Stapells (2006)	14	22.1	8.0	22.1	[17.9; 26.3]
Small et al. (2007)	10	13.0	11.6	13.0	[5.8; 20.2]
Small et al. (2007)	15	33.3	15.0	33.3	[25.7; 40.9]
Small et al. (2007)	13	16.2	9.6	16.2	[11.0; 21.4]
Small and Stapells (2008a)	35	13.9	10.5	13.9	[10.4; 17.3]
Small and Stapells (2008a)	13	13.4	9.5	13.4	[8.2; 18.5]
Small and Stapells (2008b)	12	9.2	7.9	9.2	[4.7; 13.7]
Small and Hu (2011)	22	21.1	11.0	21.1	[16.5; 25.7]
Small and Hu (2011)	10	19.6	10.8	19.6	[12.9; 26.3]
Casey and Small (2014)	20	14.5	11.4	14.5	[9.5; 19.5]
**Random Effect model**	**193**			**18.9**	**[14.3; 23.4]**

Table S7: BC ASSR thresholds in NH infants, excluding studies in children (sensitivity analysis). SD: standard deviation; CI: confident intervals.

Study	ASSR (dB HL) Total	Mean	SD	Mean (dB HL)	95%-CI
Swanepoel et al. (2008)	35.0	19.4	8.5	19.4	[16.6; 22.2]
Ismaila et al. (2016)	35.0	24.0	6.4	24.0	[21.9; 26.1]
Valeriote and Small (2024)	15.0	15.3	12.6	15.3	[8.9; 21.7]
**Random Effect model**	**85**			**20.3**	**[15.6; 24.9]**

Table S8: BC ASSR thresholds in CHL infants at 500 Hz. SD: standard deviation; CI: confident intervals.

D Spurious (non-auditory) responses

Study	Note
Small and Stapells (2005)	The percentage of participants who had thresholds ≤20 dB HL were 60, 40, 90, and 60% at 500, 1000, 2000, and 4000 Hz, respectively. The percentage of participants who had thresholds ≤30 dB HL were 90, 70, 100, and 100% at 500, 1000, 2000, and 4000 Hz, respectively.
Small and Stapells (2006)	For the post-term infants, 90% or more of the subjects had responses present at minimum intensities of 30, 10, 40, and 30 dB HL for 500, 1000, 2000, and 4000 Hz, respectively. For the preterm infants, 90% or more of the subjects had responses present at minimum intensities of 40, 30, 50, and 50 dB HL for 500, 1000, 2000, and 4000 Hz, respectively.
Swanepoel et al. (2008)	Spurious BC responses for children with severe-to-profound SNHL. See their Table 4 for % spurious for children with mild-to-moderate SNHL. Min levels where spurious results were seen: 40 dB HL @ 500 Hz; 60 dB HL @ 1000, 2000, 4000.
Ismaila et al. (2016)	Min levels where spurious results were seen: 52 dB HL @ 500 Hz; 66.5 dB HL @ 1000 Hz; 69 dB HL @ 2000 Hz; 64 dB HL @ 4000 Hz.

Table S9: Spurious (non-auditory) responses reported in the included papers. Only studies that explicitly reported spurious responses are included. SNHL: sensorineural hearing loss.

E Data

Paper	N	AgeMean	AgeMetric	AgeGroup	HearingGroup	Test	Metric	Freq	Thr
Lins et al (1996)	6.00		year	Adults	NH	B	M	500	16.00
Lins et al (1996)	6.00		year	Adults	NH	B	M	1000	13.00
Lins et al (1996)	6.00		year	Adults	NH	B	M	2000	24.00
Lins et al (1996)	6.00		year	Adults	NH	B	M	4000	16.00
Lins et al (1996)	6.00		year	Adults	NH	B	S	500	8.00
Lins et al (1996)	6.00		year	Adults	NH	B	S	1000	5.00
Lins et al (1996)	6.00		year	Adults	NH	B	S	2000	4.00
Lins et al (1996)	6.00		year	Adults	NH	B	S	4000	4.00
Lins et al (1996)	6.00		year	Adults	NH	A	M	500	26.00
Lins et al (1996)	6.00		year	Adults	NH	A	M	1000	28.00
Lins et al (1996)	6.00		year	Adults	NH	A	M	2000	33.00
Lins et al (1996)	6.00		year	Adults	NH	A	M	4000	26.00
Lins et al (1996)	6.00		year	Adults	NH	A	S	500	6.00
Lins et al (1996)	6.00		year	Adults	NH	A	S	1000	10.00
Lins et al (1996)	6.00		year	Adults	NH	A	S	2000	7.00
Lins et al (1996)	6.00		year	Adults	NH	A	S	4000	11.00
Small & Stapells (2005)	10.00		year	Adults	NH	B	M	500	4.90
Small & Stapells (2005)	10.00		year	Adults	NH	B	S	500	7.70
Small & Stapells (2005)	10.00		year	Adults	NH	B	M	1000	-3.10
Small & Stapells (2005)	10.00		year	Adults	NH	B	S	1000	4.90
Small & Stapells (2005)	10.00		year	Adults	NH	B	M	2000	4.40
Small & Stapells (2005)	10.00		year	Adults	NH	B	S	2000	6.30
Small & Stapells (2005)	10.00		year	Adults	NH	B	M	4000	-3.60
Small & Stapells (2005)	10.00		year	Adults	NH	B	S	4000	6.30
Small & Stapells (2005)	10.00		year	Adults	NH	A	M	500	22.00
Small & Stapells (2005)	10.00		year	Adults	NH	A	S	500	11.40
Small & Stapells (2005)	10.00		year	Adults	NH	A	M	1000	26.00
Small & Stapells (2005)	10.00		year	Adults	NH	A	S	1000	13.50
Small & Stapells (2005)	10.00		year	Adults	NH	A	M	2000	18.00
Small & Stapells (2005)	10.00		year	Adults	NH	A	S	2000	7.90
Small & Stapells (2005)	10.00		year	Adults	NH	A	M	4000	18.00
Small & Stapells (2005)	10.00		year	Adults	NH	A	S	4000	11.40
Small & Stapells (2006)	29.00	34.50	wk pca	Infants	NH	A	M	500	16.20
Small & Stapells (2006)	29.00	34.50	wk pca	Infants	NH	A	S	500	10.80
Small & Stapells (2006)	29.00	34.50	wk pca	Infants	NH	A	M	1000	15.50
Small & Stapells (2006)	29.00	34.50	wk pca	Infants	NH	A	S	1000	10.20
Small & Stapells (2006)	29.00	34.50	wk pca	Infants	NH	A	M	2000	37.30
Small & Stapells (2006)	29.00	34.50	wk pca	Infants	NH	A	S	2000	15.90
Small & Stapells (2006)	29.00	34.50	wk pca	Infants	NH	A	M	4000	32.50
Small & Stapells (2006)	29.00	34.50	wk pca	Infants	NH	A	S	4000	12.70
Small & Stapells (2006)	14.00	17.00	week	Infants	NH	A	M	500	13.60
Small & Stapells (2006)	14.00	17.00	week	Infants	NH	A	S	500	13.40
Small & Stapells (2006)	14.00	17.00	week	Infants	NH	A	M	1000	2.10
Small & Stapells (2006)	14.00	17.00	week	Infants	NH	A	S	1000	7.00
Small & Stapells (2006)	14.00	17.00	week	Infants	NH	A	M	2000	26.40
Small & Stapells (2006)	14.00	17.00	week	Infants	NH	A	S	2000	6.30
Small & Stapells (2006)	14.00	17.00	week	Infants	NH	A	M	4000	22.10
Small & Stapells (2006)	14.00	17.00	week	Infants	NH	A	S	4000	8.00
Small et al (2007)	10.00	17.00	week	Infants	NH	A	M	500	14.00
Small et al (2007)	10.00	17.00	week	Infants	NH	A	S	500	14.30
Small et al (2007)	10.00	17.00	week	Infants	NH	A	M	1000	6.00
Small et al (2007)	10.00	17.00	week	Infants	NH	A	S	1000	8.43
Small et al (2007)	10.00	17.00	week	Infants	NH	A	M	2000	26.00
Small et al (2007)	10.00	17.00	week	Infants	NH	A	S	2000	9.70
Small et al (2007)	10.00	17.00	week	Infants	NH	A	M	4000	13.00
Small et al (2007)	10.00	17.00	week	Infants	NH	A	S	4000	11.60
Small et al (2007)	15.00	34.50	wk pca	Infants	NH	A	M	500	16.00
Small et al (2007)	15.00	34.50	wk pca	Infants	NH	A	S	500	11.80
Small et al (2007)	15.00	34.50	wk pca	Infants	NH	A	M	1000	16.70
Small et al (2007)	15.00	34.50	wk pca	Infants	NH	A	S	1000	9.00
Small et al (2007)	13.00	34.50	wk pca	Infants	NH	A	M	2000	34.60
Small et al (2007)	13.00	34.50	wk pca	Infants	NH	A	S	2000	15.10
Small et al (2007)	15.00	34.50	wk pca	Infants	NH	A	M	4000	33.30
Small et al (2007)	15.00	34.50	wk pca	Infants	NH	A	S	4000	15.00
Small et al (2007)	13.00	15.00	week	Infants	NH	A	M	500	18.50
Small et al (2007)	13.00	15.00	week	Infants	NH	A	S	500	14.10
Small et al (2007)	13.00	15.00	week	Infants	NH	A	M	1000	3.10

Small et al (2007)	13.00	15.00	week	Infants	NH	A	S	1000	8.60
Small et al (2007)	13.00	15.00	week	Infants	NH	A	M	2000	30.00
Small et al (2007)	13.00	15.00	week	Infants	NH	A	S	2000	5.80
Small et al (2007)	13.00	15.00	week	Infants	NH	A	M	4000	16.20
Small et al (2007)	13.00	15.00	week	Infants	NH	A	S	4000	9.60
Small & Stapells (2008a)	35.00	16.00	week	Infants	NH	A	M	500	14.22
Small & Stapells (2008a)	35.00	16.00	week	Infants	NH	A	S	500	12.87
Small & Stapells (2008a)	35.00	16.00	week	Infants	NH	A	M	1000	5.25
Small & Stapells (2008a)	35.00	16.00	week	Infants	NH	A	S	1000	7.69
Small & Stapells (2008a)	35.00	16.00	week	Infants	NH	A	M	2000	26.61
Small & Stapells (2008a)	35.00	16.00	week	Infants	NH	A	S	2000	10.15
Small & Stapells (2008a)	35.00	16.00	week	Infants	NH	A	M	4000	13.86
Small & Stapells (2008a)	35.00	16.00	week	Infants	NH	A	S	4000	10.52
Small & Stapells (2008a)	13.00	18.20	months	Infants	NH	A	M	500	22.55
Small & Stapells (2008a)	13.00	18.20	months	Infants	NH	A	S	500	10.84
Small & Stapells (2008a)	13.00	18.20	months	Infants	NH	A	M	1000	13.32
Small & Stapells (2008a)	13.00	18.20	months	Infants	NH	A	S	1000	6.28
Small & Stapells (2008a)	13.00	18.20	months	Infants	NH	A	M	2000	26.57
Small & Stapells (2008a)	13.00	18.20	months	Infants	NH	A	S	2000	8.60
Small & Stapells (2008a)	13.00	18.20	months	Infants	NH	A	M	4000	13.38
Small & Stapells (2008a)	13.00	18.20	months	Infants	NH	A	S	4000	9.51
Small & Stapells (2008a)	18.00	22.90	year	Adults	NH	A	M	500	30.94
Small & Stapells (2008a)	18.00	22.90	year	Adults	NH	A	S	500	15.12
Small & Stapells (2008a)	18.00	22.90	year	Adults	NH	A	M	1000	24.32
Small & Stapells (2008a)	18.00	22.90	year	Adults	NH	A	S	1000	13.78
Small & Stapells (2008a)	18.00	22.90	year	Adults	NH	A	M	2000	20.54
Small & Stapells (2008a)	18.00	22.90	year	Adults	NH	A	S	2000	7.72
Small & Stapells (2008a)	18.00	22.90	year	Adults	NH	A	M	4000	16.27
Small & Stapells (2008a)	18.00	22.90	year	Adults	NH	A	S	4000	10.90
Small & Stapells (2008b)	12.00	21.00	week	Infants	NH	A	M	500	12.50
Small & Stapells (2008b)	12.00	21.00	week	Infants	NH	A	S	500	12.90
Small & Stapells (2008b)	12.00	21.00	week	Infants	NH	A	M	1000	5.00
Small & Stapells (2008b)	12.00	21.00	week	Infants	NH	A	S	1000	5.20
Small & Stapells (2008b)	12.00	21.00	week	Infants	NH	A	M	2000	20.00
Small & Stapells (2008b)	12.00	21.00	week	Infants	NH	A	S	2000	12.80
Small & Stapells (2008b)	12.00	21.00	week	Infants	NH	A	M	4000	9.20
Small & Stapells (2008b)	12.00	21.00	week	Infants	NH	A	S	4000	7.90
Small & Stapells (2008b)	8.00	23.00	year	Adults	NH	A	M	500	31.30
Small & Stapells (2008b)	8.00	23.00	year	Adults	NH	A	S	500	6.40
Small & Stapells (2008b)	8.00	23.00	year	Adults	NH	A	M	1000	17.50
Small & Stapells (2008b)	8.00	23.00	year	Adults	NH	A	S	1000	12.80
Small & Stapells (2008b)	8.00	23.00	year	Adults	NH	A	M	2000	20.00
Small & Stapells (2008b)	8.00	23.00	year	Adults	NH	A	S	2000	7.60
Small & Stapells (2008b)	8.00	23.00	year	Adults	NH	A	M	4000	10.00
Small & Stapells (2008b)	8.00	23.00	year	Adults	NH	A	S	4000	10.70
Swanepoel et al (2008)	23.00	2.20	year	Infants	SNHL	A	M	500	53.00
Swanepoel et al (2008)	23.00	2.20	year	Infants	SNHL	A	S	500	4.90
Swanepoel et al (2008)	23.00	2.20	year	Infants	SNHL	A	M	1000	61.10
Swanepoel et al (2008)	23.00	2.20	year	Infants	SNHL	A	S	1000	2.10
Swanepoel et al (2008)	23.00	2.20	year	Infants	SNHL	A	M	2000	68.00
Swanepoel et al (2008)	23.00	2.20	year	Infants	SNHL	A	S	2000	3.60
Swanepoel et al (2008)	22.00	2.20	year	Infants	SNHL	A	M	4000	68.40
Swanepoel et al (2008)	22.00	2.20	year	Infants	SNHL	A	S	4000	2.80
Swanepoel et al (2008)	21.00	3.60	year	Infants	NH	A	M	500	17.90
Swanepoel et al (2008)	21.00	3.60	year	Infants	NH	A	S	500	6.80
Swanepoel et al (2008)	21.00	3.60	year	Infants	NH	A	M	1000	16.00
Swanepoel et al (2008)	21.00	3.60	year	Infants	NH	A	S	1000	11.40
Swanepoel et al (2008)	21.00	3.60	year	Infants	NH	A	M	2000	23.60
Swanepoel et al (2008)	21.00	3.60	year	Infants	NH	A	S	2000	6.50
Swanepoel et al (2008)	21.00	3.60	year	Infants	NH	A	M	4000	25.50
Swanepoel et al (2008)	21.00	3.60	year	Infants	NH	A	S	4000	7.60
Swanepoel et al (2008)	13.00	2.50	year	Infants	SNHL	A	M	500	36.50
Swanepoel et al (2008)	13.00	2.50	year	Infants	SNHL	A	S	500	6.60
Swanepoel et al (2008)	13.00	2.50	year	Infants	SNHL	A	M	1000	41.50
Swanepoel et al (2008)	13.00	2.50	year	Infants	SNHL	A	S	1000	9.90
Swanepoel et al (2008)	13.00	2.50	year	Infants	SNHL	A	M	2000	56.50
Swanepoel et al (2008)	13.00	2.50	year	Infants	SNHL	A	S	2000	10.90
Swanepoel et al (2008)	13.00	2.50	year	Infants	SNHL	A	M	4000	55.40
Swanepoel et al (2008)	13.00	2.50	year	Infants	SNHL	A	S	4000	13.00
Swanepoel et al (2008)	35.00	2.70	year	Infants	CHL	A	M	500	19.40
Swanepoel et al (2008)	35.00	2.70	year	Infants	CHL	A	S	500	8.50
Swanepoel et al (2008)	35.00	2.70	year	Infants	CHL	A	M	1000	25.00

Swanepoel et al (2008)	35.00	2.70	year	Infants	CHL	A	S	1000	11.30
Swanepoel et al (2008)	35.00	2.70	year	Infants	CHL	A	M	2000	24.30
Swanepoel et al (2008)	35.00	2.70	year	Infants	CHL	A	S	2000	8.50
Swanepoel et al (2008)	35.00	2.70	year	Infants	CHL	A	M	4000	25.30
Swanepoel et al (2008)	35.00	2.70	year	Infants	CHL	A	S	4000	11.90
Ishida et al (2011)	14.00	26.80	year	Adults	NH	B	M	500	1.10
Ishida et al (2011)	14.00	26.80	year	Adults	NH	B	S	500	8.40
Ishida et al (2011)	15.00	26.80	year	Adults	NH	B	M	1000	1.70
Ishida et al (2011)	15.00	26.80	year	Adults	NH	B	S	1000	5.90
Ishida et al (2011)	15.00	26.80	year	Adults	NH	B	M	2000	9.30
Ishida et al (2011)	15.00	26.80	year	Adults	NH	B	S	2000	6.50
Ishida et al (2011)	16.00	26.80	year	Adults	NH	B	M	4000	14.40
Ishida et al (2011)	16.00	26.80	year	Adults	NH	B	S	4000	6.00
Ishida et al (2011)	14.00	26.80	year	Adults	NH	A	M	500	20.70
Ishida et al (2011)	14.00	26.80	year	Adults	NH	A	S	500	10.70
Ishida et al (2011)	15.00	26.80	year	Adults	NH	A	M	1000	14.00
Ishida et al (2011)	15.00	26.80	year	Adults	NH	A	S	1000	6.30
Ishida et al (2011)	15.00	26.80	year	Adults	NH	A	M	2000	22.00
Ishida et al (2011)	15.00	26.80	year	Adults	NH	A	S	2000	7.80
Ishida et al (2011)	16.00	26.80	year	Adults	NH	A	M	4000	27.50
Ishida et al (2011)	16.00	26.80	year	Adults	NH	A	S	4000	8.60
Ishida et al (2011)	10.00	24.60	year	Adults	NH	B	M	500	8.00
Ishida et al (2011)	10.00	24.60	year	Adults	NH	B	S	500	5.40
Ishida et al (2011)	10.00	24.60	year	Adults	NH	B	M	1000	4.00
Ishida et al (2011)	10.00	24.60	year	Adults	NH	B	S	1000	3.90
Ishida et al (2011)	10.00	24.60	year	Adults	NH	B	M	2000	5.00
Ishida et al (2011)	10.00	24.60	year	Adults	NH	B	S	2000	5.30
Ishida et al (2011)	10.00	24.60	year	Adults	NH	B	M	4000	3.00
Ishida et al (2011)	10.00	24.60	year	Adults	NH	B	S	4000	6.30
Ishida et al (2011)	10.00	24.60	year	Adults	NH	A	M	500	21.70
Ishida et al (2011)	10.00	24.60	year	Adults	NH	A	S	500	9.40
Ishida et al (2011)	10.00	24.60	year	Adults	NH	A	M	1000	15.60
Ishida et al (2011)	10.00	24.60	year	Adults	NH	A	S	1000	12.90
Ishida et al (2011)	10.00	24.60	year	Adults	NH	A	M	2000	15.90
Ishida et al (2011)	10.00	24.60	year	Adults	NH	A	S	2000	9.50
Ishida et al (2011)	10.00	24.60	year	Adults	NH	A	M	4000	11.00
Ishida et al (2011)	10.00	24.60	year	Adults	NH	A	S	4000	3.20
Ishida et al (2011)	39.00	46.00	year	Adults	SNHL	B	M	500	31.80
Ishida et al (2011)	39.00	46.00	year	Adults	SNHL	B	S	500	15.70
Ishida et al (2011)	39.00	46.00	year	Adults	SNHL	B	M	1000	34.10
Ishida et al (2011)	39.00	46.00	year	Adults	SNHL	B	S	1000	17.40
Ishida et al (2011)	39.00	46.00	year	Adults	SNHL	B	M	2000	43.50
Ishida et al (2011)	39.00	46.00	year	Adults	SNHL	B	S	2000	14.70
Ishida et al (2011)	39.00	46.00	year	Adults	SNHL	B	M	4000	48.20
Ishida et al (2011)	39.00	46.00	year	Adults	SNHL	B	S	4000	13.70
Ishida et al (2011)	39.00	46.00	year	Adults	SNHL	A	M	500	48.70
Ishida et al (2011)	39.00	46.00	year	Adults	SNHL	A	S	500	12.80
Ishida et al (2011)	39.00	46.00	year	Adults	SNHL	A	M	1000	44.60
Ishida et al (2011)	39.00	46.00	year	Adults	SNHL	A	S	1000	14.10
Ishida et al (2011)	39.00	46.00	year	Adults	SNHL	A	M	2000	52.60
Ishida et al (2011)	39.00	46.00	year	Adults	SNHL	A	S	2000	12.30
Ishida et al (2011)	39.00	46.00	year	Adults	SNHL	A	M	4000	53.10
Ishida et al (2011)	39.00	46.00	year	Adults	SNHL	A	S	4000	11.70
Small & Hu (2011)	22.00	2.10	months	Infants	NH	A	M	500	18.32
Small & Hu (2011)	22.00	2.10	months	Infants	NH	A	S	500	11.78
Small & Hu (2011)	22.00	2.10	months	Infants	NH	A	M	1000	5.71
Small & Hu (2011)	22.00	2.10	months	Infants	NH	A	S	1000	8.60
Small & Hu (2011)	22.00	2.10	months	Infants	NH	A	M	2000	28.55
Small & Hu (2011)	22.00	2.10	months	Infants	NH	A	S	2000	8.90
Small & Hu (2011)	22.00	2.10	months	Infants	NH	A	M	4000	21.14
Small & Hu (2011)	22.00	2.10	months	Infants	NH	A	S	4000	11.00
Small & Hu (2011)	10.00	15.30	months	Infants	NH	A	M	500	22.32
Small & Hu (2011)	10.00	15.30	months	Infants	NH	A	S	500	10.84
Small & Hu (2011)	10.00	15.30	months	Infants	NH	A	M	1000	16.90
Small & Hu (2011)	10.00	15.30	months	Infants	NH	A	S	1000	8.25
Small & Hu (2011)	10.00	15.30	months	Infants	NH	A	M	2000	26.99
Small & Hu (2011)	10.00	15.30	months	Infants	NH	A	S	2000	13.58
Small & Hu (2011)	10.00	15.30	months	Infants	NH	A	M	4000	19.58
Small & Hu (2011)	10.00	15.30	months	Infants	NH	A	S	4000	10.77
Small & Hu (2011)	20.00	26.50	year	Adults	NH	B	M	500	-0.70
Small & Hu (2011)	20.00	26.50	year	Adults	NH	B	S	500	6.60
Small & Hu (2011)	20.00	26.50	year	Adults	NH	B	M	1000	5.12

Small & Hu (2011)	20.00	26.50	year	Adults	NH	B	S	1000	5.65
Small & Hu (2011)	20.00	26.50	year	Adults	NH	B	M	2000	0.69
Small & Hu (2011)	20.00	26.50	year	Adults	NH	B	S	2000	5.86
Small & Hu (2011)	20.00	26.50	year	Adults	NH	B	M	4000	3.58
Small & Hu (2011)	20.00	26.50	year	Adults	NH	B	S	4000	6.08
Small & Hu (2011)	20.00	26.50	year	Adults	NH	A	M	500	23.27
Small & Hu (2011)	20.00	26.50	year	Adults	NH	A	S	500	14.14
Small & Hu (2011)	20.00	26.50	year	Adults	NH	A	M	1000	16.90
Small & Hu (2011)	20.00	26.50	year	Adults	NH	A	S	1000	12.02
Small & Hu (2011)	20.00	26.50	year	Adults	NH	A	M	2000	18.41
Small & Hu (2011)	20.00	26.50	year	Adults	NH	A	S	2000	6.55
Small & Hu (2011)	20.00	26.50	year	Adults	NH	A	M	4000	14.04
Small & Hu (2011)	20.00	26.50	year	Adults	NH	A	S	4000	9.83
Casey & Small (2014)	19.00	10.50	months	Infants	NH	B	M	500	-0.50
Casey & Small (2014)	19.00	10.50	months	Infants	NH	B	S	500	9.10
Casey & Small (2014)	4.00	10.50	months	Infants	NH	B	M	1000	2.50
Casey & Small (2014)	4.00	10.50	months	Infants	NH	B	S	1000	12.60
Casey & Small (2014)	20.00	10.50	months	Infants	NH	B	M	2000	2.50
Casey & Small (2014)	20.00	10.50	months	Infants	NH	B	S	2000	8.50
Casey & Small (2014)	18.00	10.50	months	Infants	NH	B	M	4000	5.60
Casey & Small (2014)	18.00	10.50	months	Infants	NH	B	S	4000	7.00
Casey & Small (2014)	19.00	10.50	months	Infants	NH	A	M	500	11.10
Casey & Small (2014)	19.00	10.50	months	Infants	NH	A	S	500	11.00
Casey & Small (2014)	20.00	10.50	months	Infants	NH	A	M	1000	9.00
Casey & Small (2014)	20.00	10.50	months	Infants	NH	A	S	1000	9.10
Casey & Small (2014)	20.00	10.50	months	Infants	NH	A	M	2000	20.50
Casey & Small (2014)	20.00	10.50	months	Infants	NH	A	S	2000	10.50
Casey & Small (2014)	20.00	10.50	months	Infants	NH	A	M	4000	14.50
Casey & Small (2014)	20.00	10.50	months	Infants	NH	A	S	4000	11.40
Casey & Small (2014)	8.00	29.90	year	Adults	NH	B	M	500	1.30
Casey & Small (2014)	8.00	29.90	year	Adults	NH	B	S	500	6.40
Casey & Small (2014)	8.00	29.90	year	Adults	NH	B	M	1000	1.30
Casey & Small (2014)	8.00	29.90	year	Adults	NH	B	S	1000	4.40
Casey & Small (2014)	8.00	29.90	year	Adults	NH	B	M	2000	2.50
Casey & Small (2014)	8.00	29.90	year	Adults	NH	B	S	2000	4.60
Casey & Small (2014)	8.00	29.90	year	Adults	NH	B	M	4000	0.00
Casey & Small (2014)	8.00	29.90	year	Adults	NH	B	S	4000	6.00
Casey & Small (2014)	11.00	29.90	year	Adults	NH	A	M	500	20.00
Casey & Small (2014)	11.00	29.90	year	Adults	NH	A	S	500	8.90
Casey & Small (2014)	11.00	29.90	year	Adults	NH	A	M	1000	15.50
Casey & Small (2014)	11.00	29.90	year	Adults	NH	A	S	1000	12.10
Casey & Small (2014)	11.00	29.90	year	Adults	NH	A	M	2000	12.70
Casey & Small (2014)	11.00	29.90	year	Adults	NH	A	S	2000	7.90
Casey & Small (2014)	11.00	29.90	year	Adults	NH	A	M	4000	8.50
Casey & Small (2014)	11.00	29.90	year	Adults	NH	A	S	4000	10.40
Ismaila et al (2016)	35.00	4.50	year	Infants	NH	B	M	500	13.00
Ismaila et al (2016)	35.00	4.50	year	Infants	NH	B	S	500	2.50
Ismaila et al (2016)	35.00	4.50	year	Infants	NH	B	M	1000	15.00
Ismaila et al (2016)	35.00	4.50	year	Infants	NH	B	S	1000	3.20
Ismaila et al (2016)	35.00	4.50	year	Infants	NH	B	M	2000	14.50
Ismaila et al (2016)	35.00	4.50	year	Infants	NH	B	S	2000	2.24
Ismaila et al (2016)	35.00	4.50	year	Infants	NH	B	M	4000	14.50
Ismaila et al (2016)	35.00	4.50	year	Infants	NH	B	S	4000	3.20
Ismaila et al (2016)	35.00	4.50	year	Infants	NH	A	M	500	23.50
Ismaila et al (2016)	35.00	4.50	year	Infants	NH	A	S	500	12.26
Ismaila et al (2016)	35.00	4.50	year	Infants	NH	A	M	1000	22.50
Ismaila et al (2016)	35.00	4.50	year	Infants	NH	A	S	1000	8.51
Ismaila et al (2016)	35.00	4.50	year	Infants	NH	A	M	2000	20.00
Ismaila et al (2016)	35.00	4.50	year	Infants	NH	A	S	2000	6.49
Ismaila et al (2016)	35.00	4.50	year	Infants	NH	A	M	4000	25.00
Ismaila et al (2016)	35.00	4.50	year	Infants	NH	A	S	4000	6.07
Ismaila et al (2016)	36.00	4.50	year	Infants	SNHL	B	M	500	36.25
Ismaila et al (2016)	36.00	4.50	year	Infants	SNHL	B	S	500	4.50
Ismaila et al (2016)	36.00	4.50	year	Infants	SNHL	B	M	1000	35.75
Ismaila et al (2016)	36.00	4.50	year	Infants	SNHL	B	S	1000	4.67
Ismaila et al (2016)	36.00	4.50	year	Infants	SNHL	B	M	2000	42.75
Ismaila et al (2016)	36.00	4.50	year	Infants	SNHL	B	S	2000	4.99
Ismaila et al (2016)	36.00	4.50	year	Infants	SNHL	B	M	4000	46.75
Ismaila et al (2016)	36.00	4.50	year	Infants	SNHL	B	S	4000	2.94
Ismaila et al (2016)	36.00	4.50	year	Infants	SNHL	A	M	500	52.50
Ismaila et al (2016)	36.00	4.50	year	Infants	SNHL	A	S	500	6.39
Ismaila et al (2016)	36.00	4.50	year	Infants	SNHL	A	M	1000	41.50

Ismaila et al (2016)	36.00	4.50	year	Infants	SNHL	A	S	1000	6.71
Ismaila et al (2016)	36.00	4.50	year	Infants	SNHL	A	M	2000	55.00
Ismaila et al (2016)	36.00	4.50	year	Infants	SNHL	A	S	2000	5.13
Ismaila et al (2016)	36.00	4.50	year	Infants	SNHL	A	M	4000	58.50
Ismaila et al (2016)	36.00	4.50	year	Infants	SNHL	A	S	4000	3.66
Ismaila et al (2016)	35.00	4.50	year	Infants	CHL	B	M	500	11.50
Ismaila et al (2016)	35.00	4.50	year	Infants	CHL	B	S	500	2.30
Ismaila et al (2016)	35.00	4.50	year	Infants	CHL	B	M	1000	15.00
Ismaila et al (2016)	35.00	4.50	year	Infants	CHL	B	S	1000	3.90
Ismaila et al (2016)	35.00	4.50	year	Infants	CHL	B	M	2000	14.00
Ismaila et al (2016)	35.00	4.50	year	Infants	CHL	B	S	2000	4.60
Ismaila et al (2016)	35.00	4.50	year	Infants	CHL	B	M	4000	13.50
Ismaila et al (2016)	35.00	4.50	year	Infants	CHL	B	S	4000	4.00
Ismaila et al (2016)	35.00	4.50	year	Infants	CHL	A	M	500	24.00
Ismaila et al (2016)	35.00	4.50	year	Infants	CHL	A	S	500	6.40
Ismaila et al (2016)	35.00	4.50	year	Infants	CHL	A	M	1000	23.50
Ismaila et al (2016)	35.00	4.50	year	Infants	CHL	A	S	1000	6.90
Ismaila et al (2016)	35.00	4.50	year	Infants	CHL	A	M	2000	23.50
Ismaila et al (2016)	35.00	4.50	year	Infants	CHL	A	S	2000	8.20
Ismaila et al (2016)	35.00	4.50	year	Infants	CHL	A	M	4000	22.50
Ismaila et al (2016)	35.00	4.50	year	Infants	CHL	A	S	4000	6.60
Valeriote & Small (2024)	23.00	7.36	week	Infants	NH	A	M	500	17.39
Valeriote & Small (2024)	23.00	7.36	week	Infants	NH	A	S	500	9.63
Valeriote & Small (2024)	21.00	7.36	week	Infants	NH	A	M	2000	21.00
Valeriote & Small (2024)	21.00	7.36	week	Infants	NH	A	S	2000	13.96
Valeriote & Small (2024)	15.00	6.71	week	Infants	CHL	A	M	500	15.33
Valeriote & Small (2024)	15.00	6.71	week	Infants	CHL	A	S	500	12.63

Table S10: Data. B: Behavioural BC Thresholds; A: ASSR BC Thresholds; M: Mean; S: SD; NH: Normal-hearing; SNHL: Sensorineural hearing loss; CHL: Conductive hearing loss; wk pca: post-conceptional age in weeks. Valeriote & Small (2024) reported thresholds only at 500 and 2000 Hz.

References

Brennan, S. K., Brooke, R. E., Stevens, J. C., and Brown, B. H. (2010). Effect of varying phase between frequency and amplitude modulation on bone conduction auditory steady state responses. *Ear Hear*, 31(6):815–824.

Brooke, R. E., Brennan, S. K., and Stevens, J. C. (2009). Bone conduction auditory steady state response: Investigations into reducing artifact. *Ear Hear*, 30(1):23–30.

Casey, K.-A. and Small, S. A. (2014). Comparisons of auditory steady state response and behavioral air conduction and bone conduction thresholds for infants and adults with normal hearing. *Ear Hear*, 35(4):423–439.

Çelik, O., Eskiizmir, G., and Uz, U. (2016). A Comparison of Thresholds of Auditory Steady-State Response and Auditory Brainstem Response in Healthy Term Babies. *J Int Adv Otol*, 12(3):277–281.

Cone-Wesson, B., Rickards, F., Poulis, C., Parker, J., Tan, L., and Pollard, J. (2002). The auditory steady-state response: Clinical observations and applications in infants and children. *J Am Acad Audiol*, 13(5):270–282.

Dabbous, A. O., El-Shennawy, A. M., Medhat, M. M., and Abdel-Latief, D. F. (2017). Narrow band CE-Chirp stimulus in auditory steady state response threshold estimation in normal hearers and patients with various degrees of sensorineural hearing loss. *Hearing, Balance and Communication*, 15(4):199–213.

Dabbous, A. O., Koura, R. A., and Hamdy, M. M. (2019). Comparison of thresholds estimation in adults with conductive hearing loss and normal hearing adults using auditory steady state response evoked by narrow band CE-chirps. *Hearing, Balance and Communication*, 17(1):18–26.

de Bressieux, E., Rouillon, I., Simon, F., Parodi, M., Bondi, T., and Loundon, N. (2025). ASSR and ABR tests in early diagnosis of hearing loss: A STROBE observational study. *Eur Ann Otorhinolaryngol Head Neck Dis*, pages S1879–7296(25)00137–1.

DeJonckere, P. H., Millet, B., Van Gool, R., Martens, A., and Lebacq, J. (2021). Objective frequency-specific hearing thresholds definition for medicolegal purposes in case of occupational NIHL: ASSR outperforms CERA. *J Otol*, 16(4):210–219.

D'haenens, W., Dhooge, I., Maes, L., Bockstael, A., Keppler, H., Philips, B., Swinnen, F., and Vinck, B. M. (2009). The clinical value of the multiple-frequency 80-Hz auditory steady-state response in adults with normal hearing and hearing loss. *Arch Otolaryngol Head Neck Surg*, 135(5):496–506.

Dimitrijevic, A., John, M. S., Van Roon, P., Purcell, D. W., Adamonis, J., Ostroff, J., Nedzelski, J. M., and Picton, T. W. (2002). Estimating the audiogram using multiple auditory steady-state responses. *J Am Acad Audiol*, 13(4):205–224.

Guerrero-Aranda, A., Mijares-Nodarse, E., Hernandez-Perez, H., and Torres-Fortuny, A. (2016). Confirmation of previous results of the occlusion effect through auditory steady-state responses in normal-hearing adults. *Indian Journal of Otology*, 22(4):231.

Hansen, E. E. and Small, S. A. (2012). Effective masking levels for bone conduction auditory steady state responses in infants and adults with normal hearing. *Ear Hear*, 33(2):257–266.

Ishida, I. M., Cuthbert, B. P., and Stapells, D. R. (2011). Multiple auditory steady state response thresholds to bone conduction stimuli in adults with normal and elevated thresholds. *Ear Hear*, 32(3):373–381.

Ismaila, N., El-Saiid, E., El Sebaii, A., and Fadel, H. (2016). Reliability of auditory steady-state response to bone conduction stimuli in assessing hearing loss in children. *Egypt J Otolaryngol*, 32(3):196–201.

Komazec, Z., Lemajić-Komazec, S., Jović, R., Nadj, C., Jovancević, L., and Savović, S. (2010). Comparison between auditory steady-state responses and pure-tone audiometry. *Vojnosanit Pregl*, 67(9):761–765.

Lins, O. G., Picton, T. W., Boucher, B. L., Durieux-Smith, A., Champagne, S. C., Moran, L. M., Perez-Abalo, M. C., Martin, V., and Savio, G. (1996). Frequency-specific audiometry using steady-state responses. *Ear Hear*, 17(2):81–96.

Michel, F. and Jørgensen, K. F. (2017). Comparison of threshold estimation in infants with hearing loss or normal hearing using auditory steady-state response evoked by narrow band CE-chirps and auditory brainstem response evoked by tone pips. *Int J Audiol*, 56(2):99–105.

Mo, L., Zhang, F., Han, D., and Zhang, L. (2011). Bone-conducted hearing assessment with 80-Hz multiple auditory steady-state responses to brief tones in adults with normal hearing. *ORL J Otorhinolaryngol Relat Spec*, 73(5):253–259.

Sininger, Y. S., Hunter, L. L., Hayes, D., Roush, P. A., and Uhler, K. M. (2018). Evaluation of Speed and Accuracy of Next-Generation Auditory Steady State Response and Auditory Brainstem Response Audiometry in Children With Normal Hearing and Hearing Loss. *Ear Hear*.

Small, S. A. and Hansen, E. E. (2012). Effective masking levels for bone-conducted amplitude- and frequency-modulated tones in adults with normal hearing: A behavioural study. *Int J Audiol*, 51(3):216–219.

Small, S. A., Hatton, J. L., and Stapells, D. R. (2007). Effects of bone oscillator coupling method, placement location, and occlusion on bone-conduction auditory steady-state responses in infants. *Ear Hear*, 28(1):83–98.

Small, S. A. and Hu, N. (2011). Maturation of the occlusion effect: A bone conduction auditory steady state response study in infants and adults with normal hearing. *Ear Hear*, 32(6):708–719.

Small, S. A. and Love, A. (2014). An investigation into the clinical utility of ipsilateral/contralateral asymmetries in bone-conduction auditory steady-state responses. *Int J Audiol*, 53(9):604–612.

Small, S. A., Smyth, A., and Leon, G. (2014). Effective masking levels for 500 and 2000 Hz bone conduction auditory steady state responses in infants and adults with normal hearing. *Ear Hear*, 35(1):63–71.

Small, S. A. and Stapells, D. R. (2004). Artifactual responses when recording auditory steady-state responses. *Ear Hear*, 25(6):611–623.

Small, S. A. and Stapells, D. R. (2005). Multiple auditory steady-state responses to bone-conduction stimuli in adults with normal hearing. *J Am Acad Audiol*, 16(3):172–183.

Small, S. A. and Stapells, D. R. (2006). Multiple auditory steady-state response thresholds to bone-conduction stimuli in young infants with normal hearing. *Ear Hear*, 27(3):219–228.

Small, S. A. and Stapells, D. R. (2008a). Maturation of bone conduction multiple auditory steady-state responses. *Int J Audiol*, 47(8):476–488.

Small, S. A. and Stapells, D. R. (2008b). Normal ipsilateral/contralateral asymmetries in infant multiple auditory steady-state responses to air- and bone-conduction stimuli. *Ear Hear*, 29(2):185–198.

Swanepoel, D. W., Ebrahim, S., Friedland, P., Swanepoel, A., and Pottas, L. (2008). Auditory steady-state responses to bone conduction stimuli in children with hearing loss. *Int J Pediatr Otorhinolaryngol*, 72(12):1861–1871.

Tarawneh, H. Y., Sohrabi, H. R., Mulders, W. H. A. M., Martins, R. N., and Jayakody, D. M. P. (2022). Comparison of Auditory Steady-State Responses With Conventional Audiometry in Older Adults. *Front Neurol*, 13:924096.

Torres-Fortuny, A., Hernández-Pérez, H., Ramírez, B., Alonso, I., Eimil, E., Guerrero-Aranda, A., and Mijares, E. (2016). Comparing auditory steady-state responses amplitude evoked by simultaneous air- and bone-conducted stimulation in newborns. *Int J Audiol*, 55(6):375–379.

Valeriote, H. and Small, S. A. (2024). Comparisons of Auditory Steady State and Auditory Brainstem Response Thresholds in Infants With Normal Hearing and Conductive Hearing Loss. *Canadian Journal of Speech-Language Pathology and Audiology*, 48(1):43–58.

Van Maanen, A. and Stapells, D. R. (2010). Multiple-ASSR thresholds in infants and young children with hearing loss. *J Am Acad Audiol*, 21(8):535–545.

Wang, X., Cheng, Y., Shi, J., Sheng, X., Wu, D., Zhao, Y., Li, D., He, D., and Wang, H. (2020). Comparison of auditory steady-state response and click-evoked auditory brain response in infants with different types and degrees of hearing loss. *Acta Otolaryngol*, 140(2):116–121.

Watkin, P., Tomlin, D., and Baldwin, M. (2011). Auditory steady-state responses in babies with normal hearing and with temporary conductive hearing loss. *Audiological Medicine*, 9(1):26–32.
